# Control of actin polymerization via the coincidence of phosphoinositides and high membrane curvature

**DOI:** 10.1083/jcb.201704061

**Published:** 2017-11-06

**Authors:** Frederic Daste, Astrid Walrant, Mikkel R. Holst, Jonathan R. Gadsby, Julia Mason, Ji-Eun Lee, Daniel Brook, Marcel Mettlen, Elin Larsson, Steven F. Lee, Richard Lundmark, Jennifer L. Gallop

**Affiliations:** 1 Wellcome Trust/Cancer Research UK Gurdon Institute and Department of Biochemistry, University of Cambridge, Cambridge, England, UK; 2 Department of Chemistry, University of Cambridge, Cambridge, England, UK; 3 Integrative Medical Biology, Umeå University, Umeå, Sweden; 4 University of Texas Southwestern Medical Center, Dallas, TX

## Abstract

The conditional use of actin during clathrin-mediated endocytosis in mammalian cells suggests that the cell controls whether and how actin is used. Using a combination of biochemical reconstitution and mammalian cell culture, we elucidate a mechanism by which the coincidence of PI(4,5)P_2_ and PI(3)P in a curved vesicle triggers actin polymerization. At clathrin-coated pits, PI(3)P is produced by the INPP4A hydrolysis of PI(3,4)P_2_, and this is necessary for actin-driven endocytosis. Both Cdc42⋅guanosine triphosphate and SNX9 activate N-WASP–WIP- and Arp2/3-mediated actin nucleation. Membrane curvature, PI(4,5)P_2_, and PI(3)P signals are needed for SNX9 assembly via its PX–BAR domain, whereas signaling through Cdc42 is activated by PI(4,5)P_2_ alone. INPP4A activity is stimulated by high membrane curvature and synergizes with SNX9 BAR domain binding in a process we call curvature cascade amplification. We show that the SNX9-driven actin comets that arise on human disease–associated oculocerebrorenal syndrome of Lowe (OCRL) deficiencies are reduced by inhibiting PI(3)P production, suggesting PI(3)P kinase inhibitors as a therapeutic strategy in Lowe syndrome.

## Abstract (precis)

How the membrane environment informs when and where actin is polymerized in clathrin-mediated endocytosis is unclear. Daste et al. show that high membrane curvature stimulates PI(3,4)P_2_ dephosphorylation by INPP4A and that PI(3)P recruits SNX9 in conjunction with both PI(4,5)P_2_ and high membrane curvature. Furthermore, they find that Lowe syndrome mimics this membrane microenvironment with the aberrant formation of a PI(4,5)P_2_/PI(3)P intermediate, giving rise to actin comets.

## Introduction

Actin dynamics are essential for the eukaryotic cell in providing a framework for organizing the membrane and producing force for vesicle deformation, transport of endocytic vesicles, cytokinesis, and cell protrusions, among other functions. How the array of actin nucleators, elongators, and bundlers work together to orchestrate actin assembly when and where it is needed remains a key question (Skau and Waterman, 2015). Clathrin-mediated endocytosis (CME) in mammalian cells provides a striking example of the need for regulated actin assembly.

Though it is well established that actin plays a critical role for endocytosis in yeast, where it drives inward invagination and fission of endocytic membranes (Picco et al., 2015) together with the pulling action of myosin motors (Lewellyn et al., 2015), in mammalian cells, the picture is more complicated. The degree to which actin assembly is required for CME differs depending on whether the clathrin-coated pits (CCPs) are budding from adherent, glass-facing membranes or nonadherent, media-facing areas of the cell (Yarar et al., 2005), and different cell types use actin to greater or lesser extents (Fujimoto et al., 2000; Mooren et al., 2012). One explanation is that the force from actin polymerization is used to aid deformation and scission of the vesicle when membrane tension is high (Aghamohammadzadeh and Ayscough, 2009; Ferguson et al., 2009; Boulant et al., 2011). How this tension might be sensed to trigger actin assembly is unknown.

**Figure 1.**
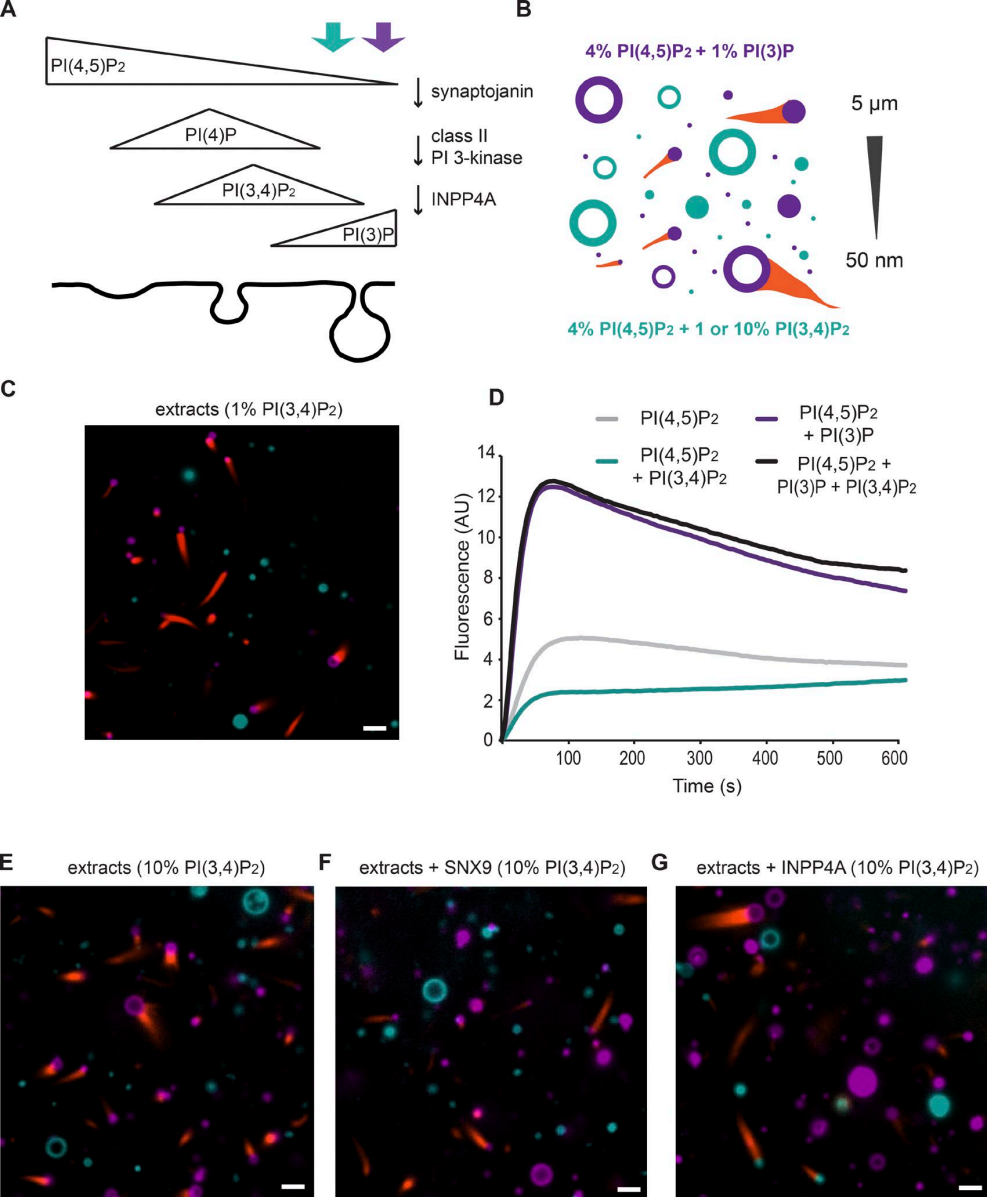
**PI(4,5)P_2_ and PI(3)P signal actin polymerization via SNX9.** (A) Cascade of phosphoinositide lipid conversion steps during endocytosis: dephosphorylation of PI(4,5)P_2_ to PI(4)P by synaptojanin, phosphorylation by PI 3-kinase to produce PI(3,4)P_2_, and dephosphorylation by INPP4A to form the PI(3)P signal characteristic of the early endosome. (B) Schematic diagram of our cell-free assay for phosphoinositide environment in actin polymerization. Competitive assay that examines phosphoinositide preferences (PI(4,5)P_2_ + PI(3)P or PI(4,5)P_2_ + PI(3,4)P_2_) in the range of curvatures at a budding CCP. (C) Direct observation of liposome samples with a continuous size distribution from 50 nm to 5 µm. Rhodamine fluorescence was used for 4% PI(4,5)P_2_, 1% PI(3)P, 65% PC, and 30% PS liposomes (all percentages by molecular fraction, purple) and NBD for 4% PI(4,5)P_2_, 1% PI(3,4)P_2_, 65% PC, and 30% PS (cyan) using spinning-disk confocal microscopy. (D) Maximal activation of actin polymerization by liposomes containing 4% PI(4,5)P_2_, 1% PI(3)P, 48% PC, and 47% PS assayed by pyrene actin assay with HSS compared with 4% PI(4,5)P_2_ alone or 4% PI(4,5)P_2_ + 1% PI(3,4)P_2_. Data show mean of four traces from two independent experiments. AU, arbitrary units. (E) As in C, except 10% PI(3,4)P_2_ (cyan). (F) 10% PI(3,4)P_2_ liposomes (cyan), extracts + SNX9. In all experiments, we exclusively observed the formation of comet tail at the surface of PI(4,5)P_2_/PI(3)P liposomes. (G) 10% PI(3,4)P_2_ liposomes (cyan) preincubated with INPP4A. Actin polymerization occurred from the cyan liposomes. Bars, 3 µm.

Actin assembly is regulated by PI(4,5)P_2_, the major signaling lipid in the inner leaflet of the plasma membrane (Saarikangas et al., 2010; Michelot and Drubin, 2011). During endocytosis, plasma membrane PI(4,5)P_2_ is dephosphorylated by the PI(4,5)P_2_ phosphatases, synaptojanin, and oculocerebrorenal syndrome of Lowe (OCRL). The resulting PI(4)P product can be phosphorylated to PI(3,4)P_2_ by the class II PI 3-kinase, PI 3-kinase C2-α (Posor et al., 2013), and then dephosphorylated at position 4 by INPP4A, yielding PI(3)P, the hallmark of the early endosomal compartment (Shin et al., 2005). Thus, during the budding of endocytic vesicles, a cascade of phosphatidylinositol conversion steps is proposed whereby sequential dephosphorylation of PI(4,5)P_2_ to PI(4)P by synaptojanin, phosphorylation by PI 3-kinase to produce PI(3,4)P_2_, and dephosphorylation ultimately form the PI(3)P signal characteristic of the early endosome (Fig. 1 A; Posor et al., 2013). How these lipid kinases and phosphatases are controlled to ensure proper spatial and temporal regulation of this cascade remains unknown.

The importance of phosphatidylinositol metabolism in actin organization and wider cell physiology is underscored by disease-causing mutations in OCRL. Deficiencies in OCRL cause Lowe syndrome, a multisystem genetic condition characterized by congenital cataracts, intellectual disability, and kidney absorption problems (Bökenkamp and Ludwig, 2016). OCRL is recruited to CCPs at late stages of CME and remains associated with early endosomes (Ungewickell et al., 2004; Vicinanza et al., 2011). Patient-derived cells exhibit defects in actin organization and endocytosis, with aberrant actin assembly on endosomes that rocket through the cytoplasm on actin comet tails (Suchy and Nussbaum, 2002; Nández et al., 2014). Congruent with the kidney absorption defects, cultured human kidney 2 and patient proximal tubule cells display reduced trafficking through the early endosomal pathway, resulting in enlarged and PI(3)P-enriched structures that accumulate actin (Vicinanza et al., 2011).

Both PI 3-kinases and synaptojanin are activated by high membrane curvature (Hübner et al., 1998; Chang-Ileto et al., 2011). Interestingly, several actin regulators that assemble at CCPs, including the adapter proteins SNX9 and TOCA-1, contain membrane curvature-sensing BAR domains. SNX9 and TOCA-1 localize to sites of CME and are also candidates for regulating actin during endocytosis via clustering of the nucleation-promoting factor neural Wiskott-Aldrich syndrome protein (N-WASP; Lundmark and Carlsson, 2003; Ho et al., 2004; Itoh et al., 2005; Soulet et al., 2005; Tsujita et al., 2006; Yarar et al., 2007). The isolated BAR–PX domain of SNX9 binds preferentially to membranes of high curvature (Pylypenko et al., 2007), and a pathway from SNX9 to actin polymerization has been reconstituted from purified components (Yarar et al., 2008). Actin polymerization downstream of TOCA-1 is observed preferentially on larger, less curved vesicles in vitro*,* which are subsequently deformed to the desired curvature. Thus, TOCA-1–mediated actin assembly drives curvature rather than responding to it (Takano et al., 2008). Whether SNX9-mediated actin assembly is dependent on membrane curvature has not been tested.

To determine how the membrane environment informs when and where actin is polymerized, we have dissected a pathway from phosphatidylinositol conversions and high membrane curvature through to actin polymerization in endocytosis. We find that high membrane curvature stimulates PI(3,4)P_2_ dephosphorylation by INPP4A and that the resulting PI(3)P in turn recruits SNX9 in conjunction with both PI(4,5)P_2_ and high membrane curvature. This three-way coincidence detection integrates the mechanics, lipid signaling, and protein cascades of CME to ensure its efficiency. We further propose that this signaling cascade can function to trigger actin assembly in response to delays in vesicle scission. Furthermore, we show that Lowe syndrome is a disease that mimics this membrane microenvironment, with the aberrant formation of a PI(4,5)P_2_/PI(3)P intermediate giving rise to actin comets. We demonstrate that the formation of actin comets in OCRL deficiency can be alleviated by reducing PI(3)P levels using PI 3-kinase inhibitors, suggesting a possible therapeutic strategy in Lowe syndrome.

## Results

### PI(4,5)P_2_ and PI(3)P signal actin polymerization via SNX9

Our previous work showed that the coincidence of PI(4,5)P_2_, PI(3)P, and membrane curvature stimulates actin polymerization in *Xenopus* egg extracts in an SNX9-dependent manner (Gallop et al., 2013). PI(4,5)P_2_ is a well-known activator of Cdc42 and N-WASP, and in turn the Arp2/3 complex. However, SNX9 has previously been implicated as an effector of PI(3,4)P_2_ after it is produced by the sequential actions of synaptojanin and PI 3-kinase C2-α (Posor et al., 2013). Therefore, to test the roles of PI(3,4)P_2_ or PI(3)P in actin polymerization within a cytoplasmic environment, we developed a competitive assay that examines phosphatidylinositol preferences in the context of high-speed supernatant (HSS) frog egg extracts (Fig. 1 B). HSS extracts provide a rich mixture of proteins necessary for early frog development and are depleted for endogenous membranes. Vesicles that contain either PI(4,5)P_2_ plus PI(3)P or PI(4,5)P_2_ plus PI(3,4)P_2_ in a phosphatidylcholine (PC) and phosphatidylserine (PS) background were labeled with rhodamine-phosphatidylethanolamine (PE) or nitro-2-1,3-benzoxadiazol (NBD)–PE, respectively, as probes to distinguish the two vesicle populations (Fig. 1 B). To mimic the range of curvatures at a budding CCP, we prepared a continuous distribution of unilamellar vesicles for each lipid composition, spanning a size range from 50 nm to 5 µm in diameter (Fig. 1 B). The vesicles were mixed and mounted in a microscopy chamber, and HSS extracts supplemented with Alexa Fluor 647–labeled actin were added, with or without the addition of excess recombinant SNX9. We observed dense accumulations of labeled actin at the vesicle surfaces that form actin comets, leading to vesicle motility (Fig. 1 C). Interestingly, comets formed exclusively from PI(4,5)P_2_/PI(3)P vesicles (Fig. 1 C, purple vesicles). Within a competitive assay format, it is possible that the concentrations of actin regulatory proteins are limited and that PI(4,5)P_2_/PI(3)P is simply preferred, rather than there being no activity arising from PI(3,4)P_2_. To distinguish between these possibilities and to provide an independent assay format to test the activity of PI(3,4)P_2_, we performed pyrene actin assays with vesicles containing PI(4,5)P_2_ (which is needed to activate Cdc42 and N-WASP) and PI(3)P or PI(3,4)P_2_. Consistent with the results from the competition assay, supplementing PI(4,5)P_2_-containing vesicles with PI(3)P resulted in a substantial increase in both the rate and extent of actin polymerization, whereas the presence of PI(3,4)P_2_ reproducibly inhibited actin polymerization (Fig. 1 D). The inhibitory effect could be caused by PI(3,4)P_2_-binding proteins within the HSS occluding PI(4,5)P_2_-binding sites, thereby inhibiting N-WASP or Cdc42 activation. However, the presence of PI(3,4)P_2_ did not inhibit actin polymerization from PI(3)P/PI(4,5)P_2_-containing vesicles (Fig. 1 D).

Even upon incorporation of 10% PI(3,4)P_2_ and with additional SNX9, actin comets failed to form on vesicles containing PI(3,4)P_2_ (Fig. 1, E and F, cyan vesicles; >250 liposomes were analyzed for each condition). Interestingly, when excess SNX9 was added, the actin comets appeared to form preferentially on the small liposomes and always on those containing PI(4,5)P_2_ and PI(3)P (Fig. 1 F, purple vesicles).

Our in vitro findings are inconsistent with previous in vivo data suggesting that SNX9 is recruited to CCPs via its interactions with PI(3,4)P_2_ (Posor et al., 2013). To reconcile these differences, we considered that although PI(3,4)P_2_ is not a direct upstream signal for actin polymerization by SNX9 in our model system, it could be a substrate for the production of PI(3)P via the action of INPP4A. To test this possibility, and also to confirm the integrity of our PI(3,4)P_2_, we repeated the previous experiment and included a preincubation of PI(3,4)P_2_-containing liposomes with recombinant INPP4A, which may be limiting in our HSS. Confirming our hypothesis, actin comet tails then arose from both sets of vesicles that originally contained either PI(3)P or PI(3,4)P_2_ (Fig. 1 G). Therefore, rather than PI(3,4)P_2_ being a direct agonist of SNX9 (Posor et al., 2013) for actin polymerization, we show that PI(3,4)P_2_ hydrolysis provides a source of PI(3)P. Together, these data establish that SNX9 is preferentially recruited to liposomes bearing PI(4,5)P_2_ and PI(3)P where it triggers actin assembly.

### Identifying the minimum machinery required for SNX9-regulated actin assembly

**Figure 2.**
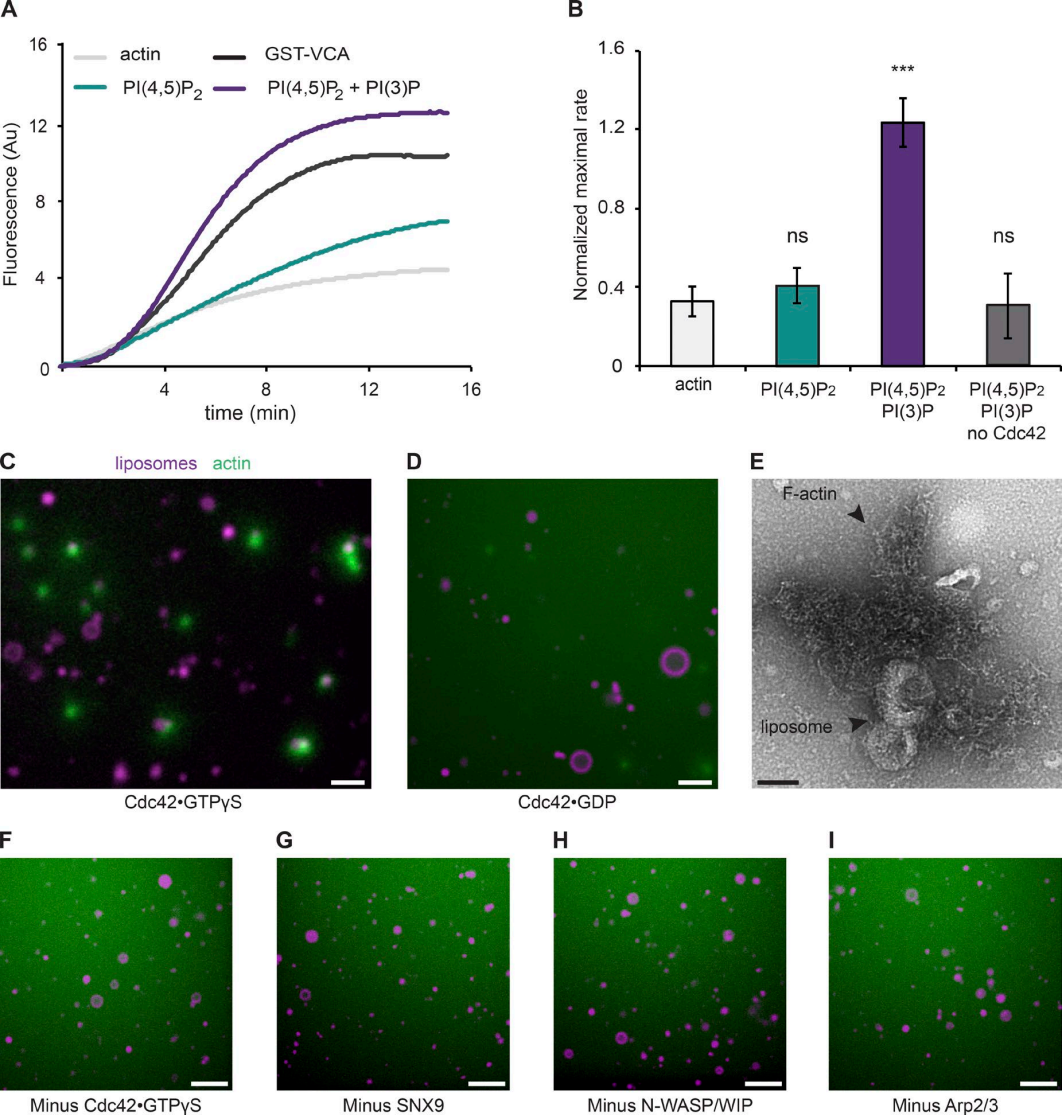
**Identifying the minimal machinery required for SNX9-regulated actin assembly.** (A) Maximal activation of actin polymerization by liposomes containing 4% PI(4,5)P_2_ + 1% PI(3)P (with 48% PC and 47% PS) assayed by pyrene actin assay in the minimal reconstituted system (20 nM Arp2/3 complex, 500 nM Cdc42⋅GTP-γS, 100 nM N-WASP/WIP complex, 100 nM SNX9, and 1 µM actin, 65:35 pyrene actin) compared with 4% PI(4,5)P_2_ alone, actin alone, and activation by GST-VCA fragment from N-WASP as a positive control. Data show the mean of eight traces. AU, arbitrary units. (B) Maximal rates from two technical repeats each of four independent experiments showing the mean and SEM. The maximal rates were normalized against 100% activation by GST-VCA. Significance was tested using an ANOVA test with a Tukey's multiple comparison post-hoc test; actin versus PI(4,5)P_2_: P = 0.900; actin versus PI(4,5)P_2_/PI(3)P: ***, P = 0.001; actin versus −Cdc42: P = 0.9000. ns, not significant. (C) Direct observation of liposomes (4% PI(4,5)P_2_, 1% PI(3)P, 65% PC, 30% PS; purple) in the presence of the minimal purified system containing 50 nM Arp2/3 complex, 50 nM Cdc42⋅GTP-γS, 100 nM N-WASP–WIP complex, 100 nM SNX9, 8 µM unlabeled actin, and 0.3 µM Alexa Fluor 647–labeled actin. Actin asters form at the surface of highly curved liposomes only when all components are present. (D) Activation of Cdc42 is needed. Direct observation of liposome samples with a continuous size distribution from 50 nm to 5 µm (4% PI(4,5)P_2_, 1% PI(3)P, 65% PC, 30% PS; purple) in the presence of the minimal purified system containing Cdc42⋅GDP. (C and D) Bars, 3 µm. (E) Electron micrograph of actin asters after incubation of PI(4,5)P_2_/PI(3)P liposomes with the minimal purified system shows disordered and branched actin filaments. Bar, 100 nm. (F–I) All components of the purified system are required for efficient actin polymerization. No actin polymerization is seen with the minimal purified system minus each individual component: Cdc42⋅GTP-γS (F), SNX9 (G), N-WASP–WIP (H), or Arp2/3 complex (I). Bars, 6 µm.

We previously identified SNX9, Cdc42⋅GTP-γS, and N-WASP as components necessary for PI(4,5)P_2_/PI(3)P-triggered actin polymerization (Gallop et al., 2013). To determine whether these factors were sufficient, we reconstituted the pathway from purified components. SNX9, Cdc42⋅GTP-γS, N-WASP (purified in its physiological complex with Wiskott-Aldrich syndrome–interacting protein [WIP], which is thought to be a more autoinhibted form; Ho et al., 2004), the Arp2/3 complex, and actin were mixed, and actin polymerization was measured using either a pyrene actin assay to quantify the rate of actin polymerization or a microscopy assay to directly visualize actin structures. In a pyrene actin assay, significant actin polymerization was only observed on vesicles containing both PI(4,5)P_2_ and PI(3)P (Fig. 2, A and B; protein purifications are shown in Fig. S1, A–E). The GST-VCA domain of N-WASP, which triggers Arp2/3 complex–mediated actin polymerization independently of Cdc42⋅GTP or BAR domain adapter protein regulation, was used as the positive control (Fig. 2 A). Unlike previous reconstitutions with SNX9 that used N-WASP alone (Yarar et al., 2008), Cdc42 was required under our assay conditions (Fig. 2 B), likely because of the use of the N-WASP–WIP complex. To confirm these findings, we visualized the polymerization of actin directly at the membrane surface via confocal imaging of PI(4,5)P_2_ plus PI(3)P vesicles (in a PC/PS background with rhodamine-PE as a probe) together with all purified components and Alexa Fluor 647–actin. The polymerization of actin over time was detected by the dense accumulation of labeled actin at the vesicle surfaces (Fig. 2 C). We also confirmed that Cdc42 must be in the GTP-bound state (Fig. 2 D), and we visualized F-actin by EM, showing the disordered nature of the actin consistent with a highly branched Arp2/3 complex–generated network (Fig. 2 E). Finally, conducting microscopy experiments in the absence of each individual component confirmed that (a) Cdc42 is essential and that (b) removal of any other component also abolishes actin polymerization (Fig. 2, F–I). Thus, PI(4,5)P_2_, PI(3)P, Cdc42⋅GTP, N-WASP–WIP, Arp2/3 complex, and SNX9 are necessary and sufficient to trigger actin polymerization.

### Synergistic binding of two phosphoinositide lipids by SNX9 subfamily PX–BAR domains

**Figure 3.**
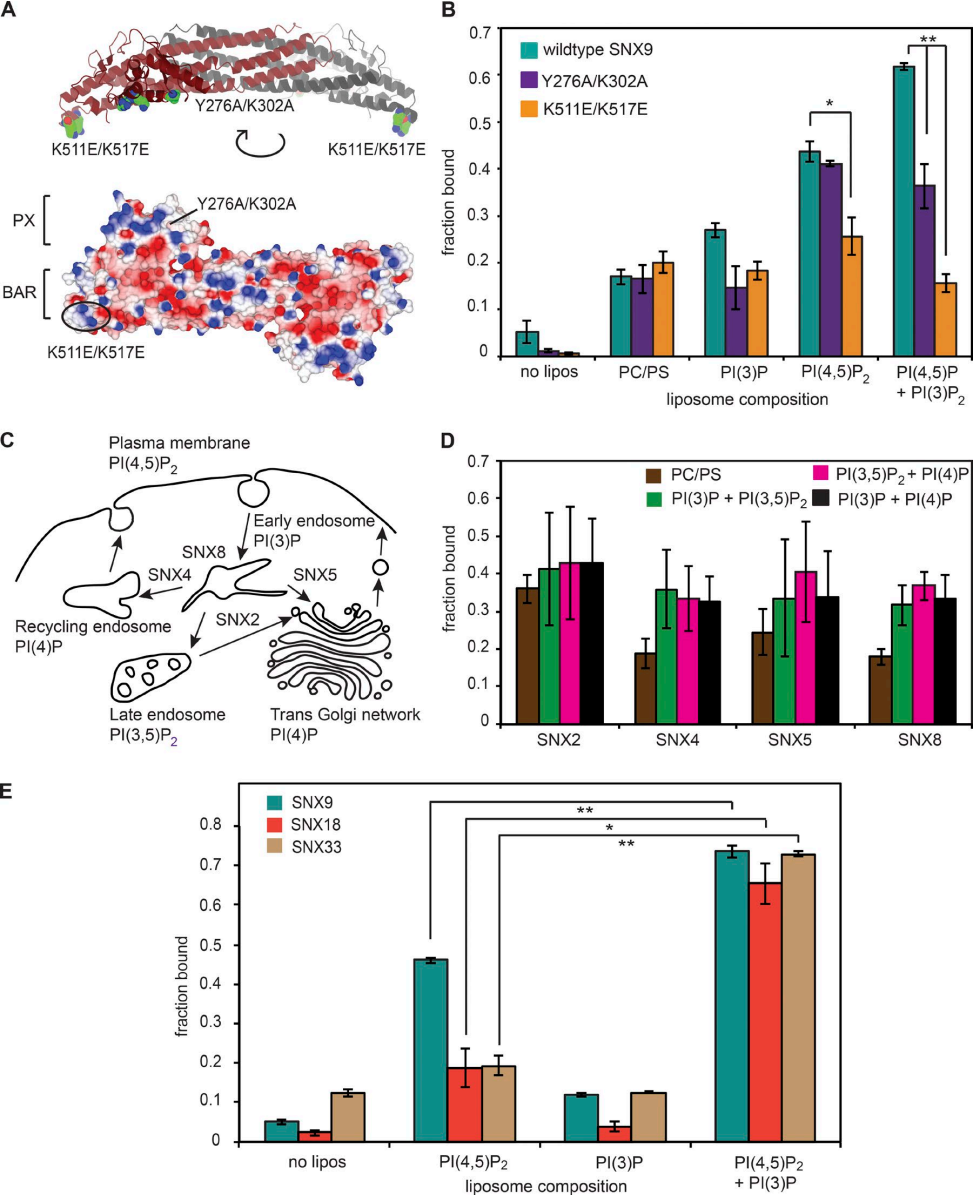
**A unique property of SNX9 subfamily in binding two phosphoinositides using two adjacent lipid-binding domains.** (A) Mutations in the various proposed lipid-binding sites within the PX and BAR domains of *Xenopus* SNX9. K511E/K517E, which are residues that make up a positively charged patch within the SNX9 BAR domain, and Y276A/K302A, which is the phosphoinositide lipid–binding pocket within the PX domain. The structure shown is 2RAI, downloaded from the Protein Data Bank and visualized in the CCP4mg molecular graphics package with the *Xenopus* numbering inferred from a sequence alignment performed in Lasergene software. (B) The liposome compositions used were 5% PI(4,5)P_2_, 5% PI(3)P, or 4% PI(4,5)P_2_/1% PI(3)P with 65% PC and 30% PS. Mutagenesis shows that binding of SNX9 to 100-nm PI(4,5)P_2_ liposomes is driven by positively charged patches on the BAR domain binding to PI(4,5)P_2_. The pocket on the PX domain is important for binding PI(4,5)P_2_ and PI(3)P. Data are the mean of three independent liposome batches and sedimentation assays. One-way ANOVA between mutants is not significant for PI(3)P: P = 0.0594. One-way ANOVA between mutants is significant for PI(4,5)P_2_: P = 0.0133; and PI(4,5)P_2_/PI(3)P: P = 9.25 × 10^−5^. By post-hoc Tukey's honest significant difference (HSD) test, the difference between WT and Y276A/K302A for PI(4,5)P_2_/PI(3)P: **, P = 0.00223; for PI(4,5)P_2_: P = 0.864; and for PI(3)P: P = 0.0730. For the WT compared with K511E/K517E mutant with PI(4,5)P_2_/PI(3)P: **, P = 0.00101; for PI(4,5)P_2_: *, P = 0.0161; and for PI(3)P: P = 0.098. (C) Phosphoinositides and SNX-BAR proteins in the endosomal network. Schematic diagram showing putative double phosphoinositide compositions for trafficking between compartments where SNX PX–BAR domains are implicated. (D) Sedimentation assay of SNX2, 4, 5, or 8 on liposomes that contain various double phosphoinositide lipid compositions. The liposome compositions were 5% of each phosphoinositide with 65% PC and 30% or 25% PS. Data are the mean of three independent liposome batches and sedimentation assays. No binding is above background suggesting that double phosphoinositide binding is a property specific to the SNX9 subfamily. (E) Sedimentation assay of extracts with liposomes that contain 4% PI(4,5)P_2_; 1% PI(3)P; or 4% PI(4,5)P_2_/1% PI(3)P and 65% PC; and 30, 34, or 31% PS with SNX9, 18, and 33 detected by Western blotting. Quantification from three independent liposome batches and Western blots. Error bars are the SEM. One-way ANOVA for SNX9 gives P = 3.113 × 10^−8^ with Tukey's post-hoc HSD test between PI(4,5)P_2_ and PI(4,5)P_2_/PI(3)P: **, P = 0.00100. One-way ANOVA for SNX18 gives P = 0.0013 with Tukey's post-hoc HSD test between PI(4,5)P_2_ and PI(4,5)P_2_/PI(3)P: *, P = 0.0112. One-way ANOVA for SNX33 gives P = 10^−6^ with Tukey's post-hoc HSD test between PI(4,5)P_2_ and PI(4,5)P_2_/PI(3)P: **, P = 0.00101.

SNX9 has previously been shown to bind promiscuously to a variety of phosphoinositide lipids (Pylypenko et al., 2007; Yarar et al., 2008), each assayed independently. To look in more detail at the apparently synergistic binding of two phosphoinositide lipids, we probed the role of the adjacent BAR and PX domains in determining the ability of SNX9 to detect the coincidence of PI(4,5)P_2_ and PI(3)P. By aligning *Xenopus* sequences with the existing crystal structure (Fig. 3 A; Pylypenko et al., 2007), we generated mutants in the proposed lipid-binding sites within the PX and BAR domains of *Xenopus* SNX9 and compared their lipid-binding specificities with the WT protein (Fig. 3 B). Two mutants were generated: K511E/K517E (*Xenopus* residue numbers), which make up a positively charged patch within the SNX9 BAR domain, and Y276A/K302A, which is present in the phosphoinositide lipid–binding pocket within the PX domain (Fig. 3 A). We first examined the binding of these mutants to PI(3)P-containing liposomes compared with WT protein (Fig. 3 B). The weak binding observed with WT SNX9 was reduced to background levels seen with PC/PS liposomes by either of the mutations (Fig. 3 B). Thus, both the PX and BAR domains contribute to weak PI(3)P binding with no apparent synergy. As previously reported for mammalian SNX9 (Pylypenko et al., 2007; Yarar et al., 2008), *Xenopus* SNX9 bound more strongly to PI(4,5)P_2_-containing liposomes. Interestingly, this binding was unaffected by the PX domain mutation but was markedly inhibited by the BAR domain mutation. When PI(4,5)P_2_ and PI(3)P were presented together, the membrane–protein interaction was of substantially higher affinity, and then both the PX phosphoinositide lipid–binding pocket and the BAR domain made significant contributions to the interaction (Fig. 3 B; gels are shown in Fig. S2 A). These data thus support a model in which the PX–BAR domain encodes a dual specificity phosphoinositide lipid–binding module that enables the coincident detection of two phosphoinositide lipids.

SNX9 is a member of a subfamily of closely related sorting nexins that includes SNX18 and 33 (Carlton et al., 2005), as well as a larger family that encodes PX–BAR domains (Worby and Dixon, 2002), but lacks the SH3 domain. These include SNX2, 4, 5, and 8, which function in trafficking from the early endosome to the TGN, plasma membrane, or late endosome (Fig. 3 C). To determine whether synergistic binding to two lipid species was a unique property of the SNX9 subfamily, or rather of all PX–BAR modules, we expressed these proteins and examined their liposome-binding properties (Fig. 3 D and Fig. S2 B). Though all the SNX PX–BARs interact with the membranes used, we could not detect specific double phosphoinositide selectivity testing against likely lipid combinations implied by their involvement in trafficking from endosomes (Fig. 3 C). In contrast, sedimentation assays and Western blotting for SNX18 and 33 showed that these SNX9 subfamily members also preferentially bound to liposomes containing both PI(4,5)P_2_ and PI(3)P (Fig. 3 E and Fig. S2 C). We verified these results using flotation assays instead of the sedimentation assay (Fig. S2, D and E). Although we have not tested all possible combinations of phosphoinositide lipids, our assays using the most likely combinations indicate that dual phosphoinositide lipid binding is likely a property unique to the SNX9, 18, and 33 subfamily of PX-BARs.

### Membrane curvature–dependent SNX9 binding and INPP4A activity combine to regulate actin polymerization

The BAR–PX domain of SNX9 has previously been reported to preferentially bind smaller, highly curved liposomes via the curvature of the BAR domain and the insertion of an amphipathic helix (Pylypenko et al., 2007). Consistent with this, we previously observed SNX9-dependent, curvature-sensitive actin polymerization on PI(4,5)P_2_/PI(3)P liposomes (Gallop et al., 2013). Close examination of the microscopy images in Fig. 1 suggested that, compared with extracts alone, there was a drive toward actin polymerization from small, diffraction-limited vesicles both when SNX9 was added and after hydrolysis of PI(3,4)P_2_ by INPP4A (Fig. 1, compare E with F and G). We therefore explored this phenomenon further to better define the mechanism for membrane curvature sensing in the PI(4,5)P_2_/PI(3)P pathway of actin polymerization.

**Figure 4.**
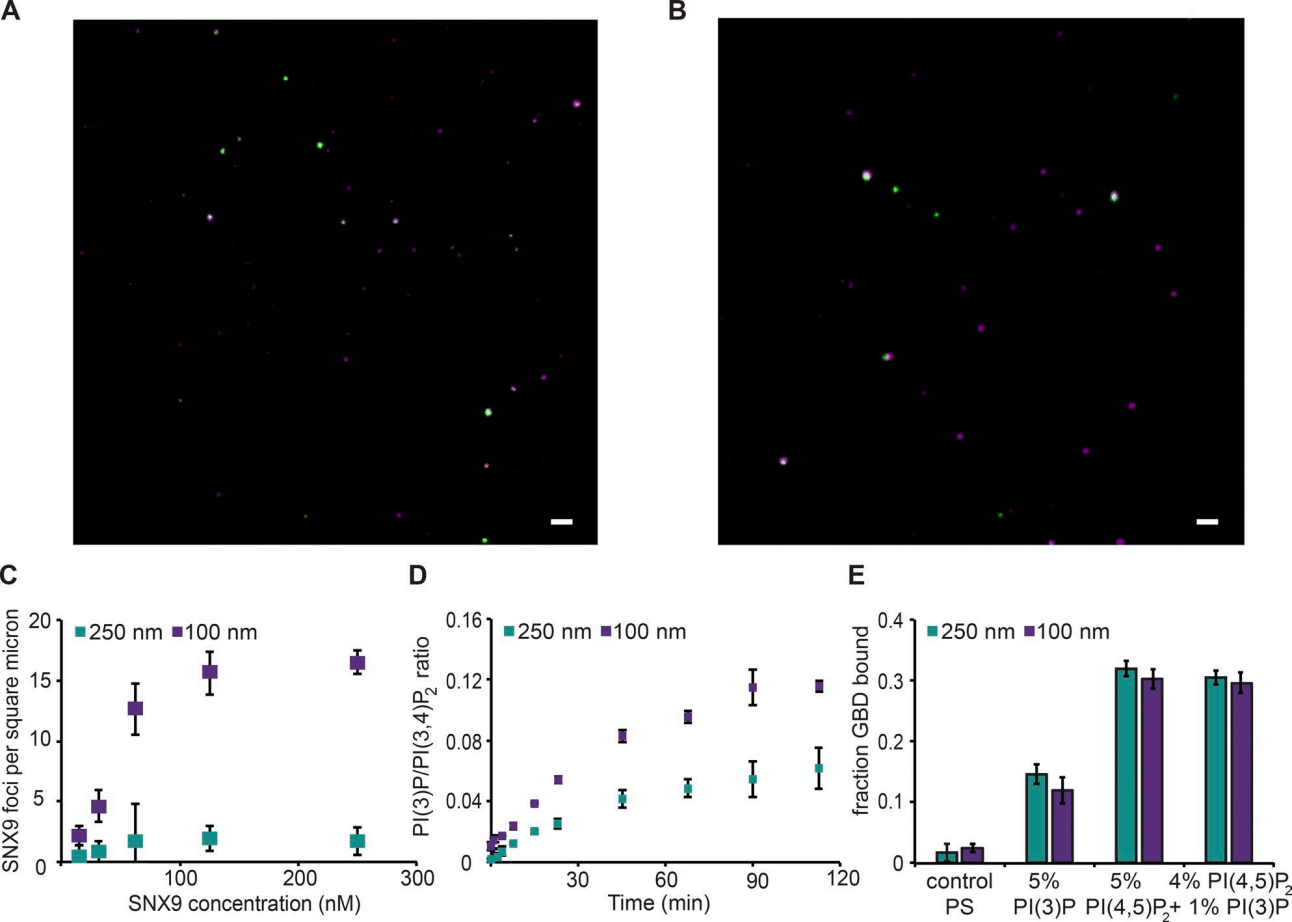
**Membrane curvature-dependent SNX9 binding and INPP4A activity combine to regulate actin polymerization.** (A and B) PI(4,5)P_2_/PI(3)P liposomes (rhodamine-PE labeled) were incubated with Alexa Fluor 647–SNAP-SNX9 from 15 to 250 nM. Concentrations and the fraction of liposomes with SNX9 foci were counted by two-color confocal imaging. Example confocal micrographs of SNX9 (green) bound to 100-nm liposomes (purple, A) and 250-nm liposomes (purple, B). Bars, 3 µm. (C) The number of SNX9 foci per surface area of membrane calculated using the means determined in Fig. S3 C. Data are from three independent experiments. (D) Kinetics of PI(3,4)P_2_ hydrolysis from 100- or 250-nm vesicles by INPP4A. Initial linear rates over the first 10 min were 0.0028 ± 0.00008 (100-nm vesicles) and 0.0013 ± 0.00021 (250-nm vesicles); P = 0.0037, *n* = 3 using Student’s *t* test. Data are from three independent experiments showing the mean and SEM. (E) Cdc42 is not regulated by membrane curvature or PI(3)P. Sedimentation assay of mKate-GBD for Cdc42⋅GTP activation on 100-nm or 250-nm vesicles (various phosphoinositide compositions, control with PC and 30% PS only) shows that Cdc42 activation is curvature independent. Quantification from three independent vesicle batches and Western blots. All error bars show SEM.

To measure the preference of SNX9 for binding small PI(4,5)P_2_/PI(3)P liposomes, we generated 100- or 250-nm mean diameter liposomes and used superresolution microscopy to directly measure their underlying size distribution. Liposomes were superresolved with point accumulation for imaging nanoscale topology (PAINT) microscopy using Nile red, and their individual diameters were measured by fitting with a two-dimensional Gaussian function (Fig. 4, A and B; Sharonov and Hochstrasser, 2006). By measuring the liposomes one by one, it is possible to directly extract both the entire size distribution and total membrane area from a liposome count (under the assumption that the liposomes are spherical; Fig. S3, A and B). Dynamic light-scattering data indicate a slightly larger size, as expected from the disproportionate effect of larger vesicles to the scattering (Fig. S3 D). To determine a binding curve, PI(4,5)P_2_/PI(3)P liposomes of each size distribution were incubated with increasing concentrations of SNX9, and the fraction of liposomes with SNX9 bound was counted by two-color confocal imaging (between 152 and 518 liposomes were counted for each condition; Fig. 4, A–C). Though measuring number alone shows some effect of curvature, saturating at 50% of liposomes bound for 100 nm and 35% bound for 250 nm (i.e., there are more binding sites for SNX9 present on more highly curved surfaces), the key measure is actually the surface area of membrane presented to a given concentration of SNX9. Because the more highly curved liposomes are smaller, less surface area of membrane is present per liposome compared with less curved ones. To adjust for this, we calculated the surface area of membrane provided by the numbers of 100- or 250-nm liposomes recorded from the confocal images based on the size distributions measured by PAINT microscopy (Fig. 4 A and Fig. S3). The SNX9 binding curve reveals an ∼10-fold increase in SNX9 binding per surface area on small liposomes (Fig. 4 C).

INPP4A is predicted to contain a double C2 domain (Ivetac et al., 2005), which is known to confer curvature sensitivity in synaptotagmin (Martens et al., 2007). We therefore assessed whether INPP4A activity exhibited curvature sensitivity. As shown in Fig. 4 D, the rate of hydrolysis of PI(3,4)P_2_ to PI(3)P by recombinant INPP4A was approximately twofold faster for 100-nm liposomes as compared with 250-nm liposomes. Thus, as was shown for other lipid phosphatases and kinases, INPP4A phosphatase activity exhibits curvature sensitivity (Fig. 4 D).

### Cdc42 guanine nucleotide exchange factor activity in extracts is not regulated by membrane curvature

Various Cdc42 guanine nucleotide exchange factors contain BAR domains and are predicted to localize to regions of high membrane curvature. To test whether the activation of Cdc42 is dependent on either membrane composition or curvature, we assayed for the presence of Cdc42⋅GTP (i.e., Cdc42 activation) on vesicles by sedimentation assays using the G protein–binding domain (GBD) from N-WASP (which binds Cdc42⋅GTP and not Cdc42⋅GDP) in the context of HSS. These experiments demonstrated that GBD recruitment occurs preferentially on PI(4,5)P_2_-containing membranes as compared with PI(3)P-containing liposomes, but without any curvature sensitivity (Fig. 4 E and Fig. S3 E). The addition of PI(3)P to PI(4,5)P_2_-containing liposomes had no further effect on Cdc42 activation. These data show that neither curvature nor synergy with PI(3)P triggers Cdc42 activation in the actin polymerization pathway; rather, Cdc42 is triggered by PI(4,5)P_2_ alone.

### Mechanism of membrane curvature–activated actin polymerization

**Figure 5.**
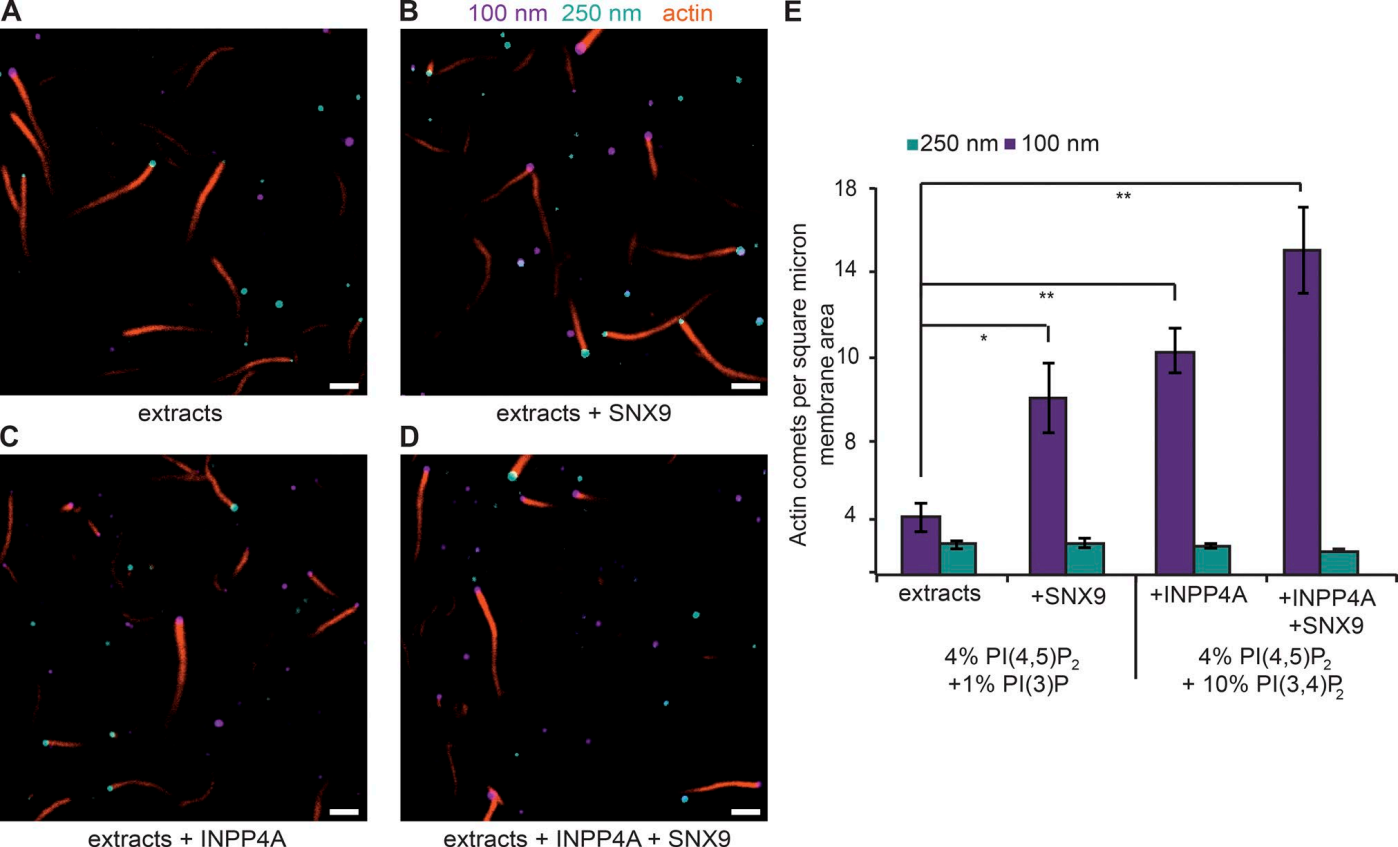
**Membrane curvature preferences in INPP4A and SNX9 propagate to actin polymerization.** Direct observation of vesicle samples containing 100-nm (rhodamine fluorescence, purple) and 250-nm (NBD, cyan) vesicles in a 1:1 ratio. (A) The lipid composition was 4% PI(4,5)P_2_, 1% PI(3)P, 65% PC, and 30% PS, and actin polymerization was triggered by the addition of HSS. (B) The lipid composition was 4% PI(4,5)P_2_, 1% PI(3)P, 65% PC, and 30% PS, and actin polymerization was triggered by the addition of HSS supplemented with 100 nM SNX9. (C) The lipid composition was 4% PI(4,5)P_2_, 10% PI(3,4)P_2_, 65% PC, and 30% PS preincubated with INPP4A. Polymerization was triggered by addition of HSS plus actin. (D) The lipid composition was 4% PI(4,5)P_2_, 10% PI(3,4)P_2_, 65% PC, and 30% PS preincubated with INPP4A. Polymerization was triggered by addition of HSS plus 100 nM SNX9 and actin. (E) Quantification of three independent experiments, 10 fields of view per condition (∼700 vesicles). One-way ANOVA between all conditions tested is significant: P = 0.0002. By post-hoc Tukey's HSD test, difference between extracts only and extracts plus SNX9: *, P = 0.040; for extracts only and extracts plus INPP4A: **, P = 0.005; and for extracts only and extracts plus INPP4A + SNX9: **, P = 0.001.

To assess the effects of the combined curvature dependence of SNX9 and INPP4A on actin polymerization on the surface of PI(4,5)P_2_/PI(3)P liposomes, we adapted our competitive microscopy assay by using 100-nm and 250-nm distributed vesicles containing rhodamine-PE or NBD-PE, respectively, so that they could be distinguished in the images. Note that the vesicles are diffraction limited and will have different fluorescent intensities depending on their position in the plane of focus as well as their size (Fig. 5, A–D). To obtain a baseline measure, we first incubated PI(4,5)P_2_/PI(3)P liposomes of both sizes with HSS (Fig. 5, A and E). The addition of SNX9 increased the number of 100-nm liposomes that had a comet tail (Fig. 5, B and E) without affecting 250-nm liposomes. This shows that the curvature selective binding of PI(3)P is transduced down the cascade of the actin regulatory network to actin polymerization itself. To assess the effect of the curvature selectivity of INPP4A, we performed the same assay with PI(4,5)P_2_/PI(3,4)P_2_ liposomes, preincubating them with INPP4A before adding extracts. Compared with PI(4,5)P_2_/PI(3)P liposomes, there was an increase in the number of comet tails from 100-nm liposomes, showing that PI(3)P production is limiting and again that the curvature sensitivity in PI(3)P formation is transduced down the cascade of the actin regulatory network (Fig. 5, C and E). Combining the effects of INPP4A activity with added SNX9 resulted in a further increase in actin polymerization on 100-nm PI(4,5)P_2_/PI(3,4)P_2_ liposomes, again without affecting polymerization on 250-nm liposomes (Fig. 5, D and E). Altogether, these results provide evidence that at least two molecular mechanisms, SNX9 binding and INPP4A activation, synergize to trigger actin polymerization at sites of high membrane curvature.

### INPP4A is important for SNX9 recruitment to CCPs, revealing CME as a functional site of PI(4,5)P_2_/PI(3)P actin polymerization

As yet, no cellular role has been proposed for the coincidence detection of two phosphoinositide lipids and membrane curvature. The important combination of signals we identified for actin polymerization in frog egg extracts is evocative of endocytosis for several reasons. First, PI(4,5)P_2_ is enriched at the plasma membrane, PI(3)P is produced at the early endosome, and membrane curvature increases during CCP maturation. Second, SNX9 is mainly recruited at the end of CCP maturation (Taylor et al., 2011; Posor et al., 2013; Schöneberg et al., 2017) and can induce actin polymerization, which has been implicated in the scission of endocytic vesicles (Lundmark and Carlsson, 2009). Finally, knockdown of the PI(3,4)P_2_ phosphatase INPP4A, which produces PI(3)P from PI(3,4)P_2_, has been seen to reduce transferrin uptake (Shin et al., 2005). Moreover, although PI(3)P is more conventionally associated with early endosomes, PI(3)P is observed at the plasma membrane (Ivetac et al., 2005; Shin et al., 2005). Thus, we hypothesized that PI(3,4)P_2_ is hydrolyzed to PI(3)P at very late stages of CCP maturation and that PI(3)P mediates SNX9 recruitment.

**Figure 6.**
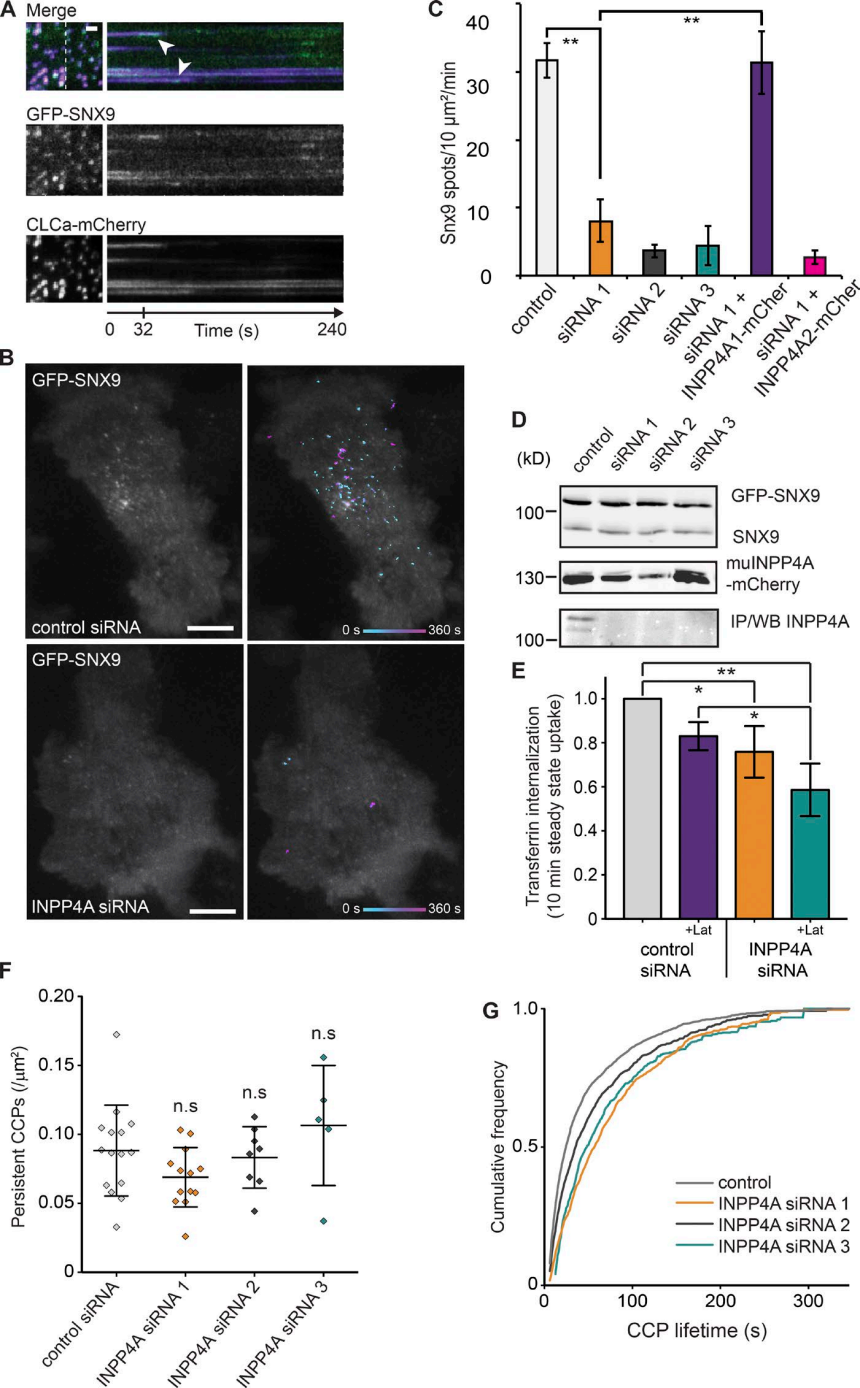
**PI(4,5)P_2_/PI(3)P stimulated actin polymerization functions during CME.** (A) TIRFM imaging (4 min) of a cell stably expressing GFP-SNX9 and transiently transfected with clathrin-Lca-mCherry. A region of interest indicated by the white dashed line is presented as a kymograph. SNX9 frequently peaks at the end of the clathrin track, indicated by white arrowheads. Bar, 1 µm. (B) HeLa cells stably expressing GFP-SNX9 are presented from single frames of TIRFM videos. Recordings of the accumulated GFP-SNX9 spots during a 4-min TIFRM time lapse are overlaid in the representative micrographs on the right. Bars, 10 µm. siRNA-mediated INPP4A knockdown decreases the number of SNX9 spots forming at the cell surface. (C) Quantification of SNX9 spot dynamics from three to eight cells per sample from three independent experiments. Student's *t* test between control and siRNA1: **, P = 0.0043. Between siRNA1 and siRNA1 + muINPP4A1-mCherry: **, P = 0.004. (D) Western blots (WB) showing that levels of GFP-SNX9 and endogenous SNX9 remain the same in INPP4A knockdown cells. Levels of INPP4A are reduced with all three siRNAs against INPP4A. INPP4A was concentrated by immunoprecipitation (IP) and then detected by Western blotting. Expression of murine INPP4A and variant1-mCherry is resistant to INPP4A siRNA1 and 3. (E) Transferrin uptake was reduced on INPP4A reduction and latrunculin A treatment. Transferrin internalization was measured without fetal bovine serum starvation (steady state) to avoid perturbation of CME. Quantification was from three independent experiments; ordinary one-way ANOVA gives P = 0.0033; Tukey's HSD test control to INPP4A siRNA: *, P = 0.0431; control to INPP4A siRNA + Lat: **, P = 0.0021; and control + Lat compared with INPP4A + Lat: *, P = 0.0408. (F) Persistent CCPs (of long duration and large size) are unaffected by INPP4A siRNA. *n* = 15 control, 13 INPP4A siRNA1-, 8 siRNA2-, and 5 siRNA3-treated cells. ns, not significant. (G) Lifetime of remaining CCPs was increased with INPP4A knockdown. Numbers of CCPs analyzed were control, 4,122; INPP4A siRNA1, 1,340; INPP4A siRNA2, 1,573; and INPP4A siRNA3, 629. Kolmogorov–Smirnov test control versus INPP4A siRNA1 is P < 0.0001, control versus INPP4A siRNA2 is P = 0.0268, and control versus INPP4A siRNA3 is P < 0.0001. Error bars show SEM.

To test whether hydrolysis of PI(3,4)P_2_ by INPP4A is important for recruitment of SNX9 and CME, HeLa cells stably expressing GFP-SNX9 at approximately twofold endogenous levels (Fig. S4, A and B), together with clathrin light chain–mCherry, were imaged using time-lapse total internal reflection fluorescence (TIRF) microscopy (TIRFM). In control conditions, these cells recapitulate the previously reported kinetics of SNX9 recruitment (Taylor et al., 2011; Posor et al., 2013), in which SNX9 colocalizes with some CCPs shortly before their disappearance (Fig. 6 A). In contrast, when levels of INPP4A are reduced using any of three separate siRNA sequences, there is a drastic drop in the number of SNX9 spots arising in the time-lapse experiments (Fig. 6 B, Fig. S4 C, and Videos 1 and 2). Quantifying the number of spots that appears per unit area showed a significant decrease (Fig. 6 C). To confirm the specificity of the RNAi treatment, we expressed siRNA-resistant murine INPP4A isoform 1 in INPP4A siRNA1–treated cells and rescued the numbers of SNX9 spots (Fig. 6 C and Fig. S4 D). Expression of INPP4A isoform 2, which is missing the N-terminal C2 domain, failed to rescue SNX9 assembly (Fig. 6 C). At the few CCPs that recruit SNX9 under INPP4A knockdown conditions, the SNX9 foci persisted for the same time as in WT cells, showing that INPP4A regulates recruitment rather than SNX9 lifetime at CCPs (Fig. S4 E). Western blotting confirmed INPP4A depletion for each siRNA sequence and that endogenous and GFP-tagged SNX9 levels remained the same under all conditions (Fig. 6 D).

The extent to which CME depends on actin is variable between cell types and conditions and remains poorly understood. We measured the rate of transferrin uptake and found that INPP4A knockdown resulted in a modest but significant decrease (Fig. 6 E). Interestingly, treatment of these cells with latrunculin A inhibited transferrin to a similar extent, whereas combining INPP4A knockdown and latrunculin A treatment appeared to compound the effect (Fig. 6 E). As these interventions are not complete, these data could suggest either that the two perturbations can both contribute to a single process or that there are INPP4A-independent ways in which actin is contributing to endocytosis. To measure the effects of INPP4A knockdown on the rate of CCP maturation, we quantified the dynamic, diffraction-limited CCPs using previously described software (Aguet et al., 2013). As expected for HeLa cells as well as the larger clathrin plaques, numerous clathrin spots within the cells remain for a duration longer than the length of the video. The numbers of these persistent CCPs were unaffected by INPP4A siRNA (Fig. 6 F). However, the mean lifetime of the bona fide CCPs was increased (Fig. 6 G) from a mean of 24.3 s in control to 56.4 s for INPP4A siRNA1, 35.9 s for siRNA2, and 47.9 s for siRNA3. These lifetime data are consistent with the inhibition of CME we detected by transferrin uptake. Thus, inhibiting the conversion of PI(3,4)P_2_ to PI(3)P inhibits the recruitment of BAR domain protein SNX9 to CCPs and decreases the efficiency of CME.

### PI(3)P production and the INPP4A–SNX9 pathway are needed for actin comets in OCRL deficiency

The activity and recruitment of PI 5-phosphatase OCRL to very early endosomes to degrade PI(4,5)P_2_ is highly regulated by membrane curvature, protein–protein interactions, and Rab35 (Billcliff et al., 2016; Cauvin et al., 2016). SNX9 is also reported to directly interact with the OCRL (Nández et al., 2014), suggesting a role in rocketing early endosomal intermediates into the cell. At the cellular level, mutations in OCRL, which cause Lowe syndrome and the less severe Dent-2 disease, disrupt actin organization and lead to the formation of SNX9-mediated comet tails propelling clathrin-coated vesicles through the cytosol in patient fibroblasts (Nández et al., 2014). We therefore surmised that the defects in OCRL may be caused by dysregulation of the SNX9 actin assembly pathway defined herein and therefore could be disrupted by inhibiting PI(3)P production.

**Figure 7.**
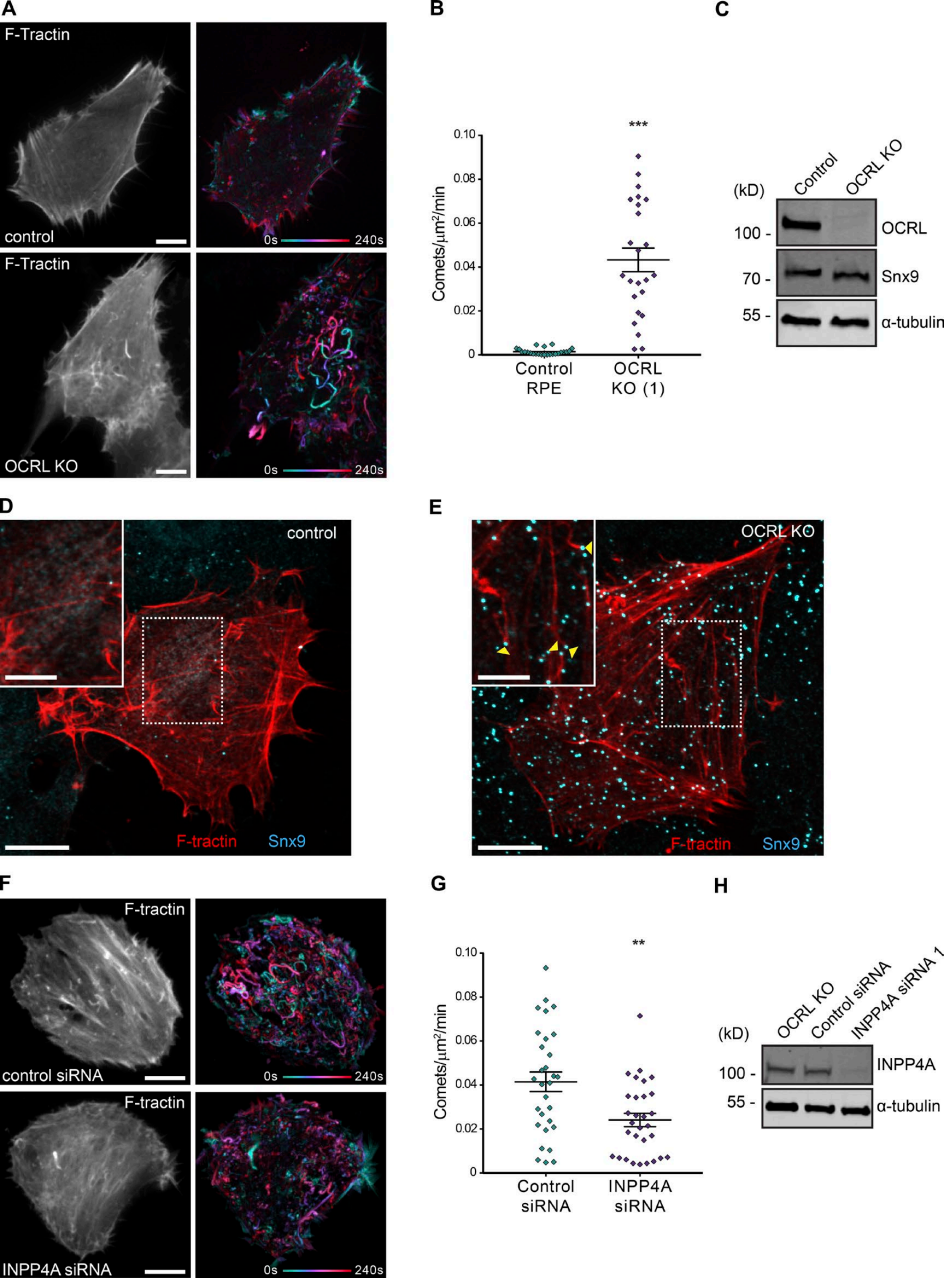
**Actin comets in OCRL-deficient RPE cells and their reduction on INPP4A siRNA.** (A) Live imaging over a 4-min period of F-tractin expressing control RPE and OCRL KO cells as shown in Video 3, showing motile actin comets in the KO not observed in control cells. The single channel image indicates a single time point, and the colored image shows a projection of the entire time course, temporally colored as indicated. (B) Quantification of actin comets from *n* = 24 cell regions for each condition. Each data point is plotted individually, along with the mean ± SEM for each condition. Difference assessed by Student’s *t* test. ***, P < 0.0001. (C) Western blot confirming OCRL KO in the OCRL KO CRISPR/Cas9 edited clone. Levels of SNX9 remain unchanged. α-Tubulin acts as a loading control. (D and E) Control and OCRL KO cells expressing GFP–F-tractin and immunolabeled for SNX9 with enlarged regions as insets. SNX9 puncta are always located at the head of actin comets (arrowheads in E). (F) Live imaging over a 4-min period of F-tractin OCRL KO cells treated with nontargeting control siRNA or INPP4A siRNA1, as shown in Video 5, showing reduction in comets on INPP4A knockdown. Bars: (main images) 10 µm; (insets) 5 µm. (G) Quantification of actin comets from *n* = 30 cell regions for each treatment. Bars indicate the mean ± SEM for each condition. Difference assessed by Student’s *t* test. **, P = 0.002. (H) Western blot verifies INPP4A knockdown in the OCRL KO clone.

As an almost complete depletion of OCRL expression is required to manifest endocytic defects (Vicinanza et al., 2011) and as PI(4,5)P_2_ enrichment alone might also be expected to cause actin comets (by recruiting SNX9 and Cdc42), we sought a complete deficiency while also controlling the genetic background of cells to create control and OCRL-deficient cells that were perfectly matched. We made retinal pigmented epithelial (RPE-1) cells deficient in OCRL and similar to mutations that occur in Lowe syndrome by targeting exon 9 using CRISPR-Cas9^D10A^ nickase–based genome editing (Fig. S5, A–D). The OCRL-deficient cells recapitulated the actin comet phenotype previously characterized in patient fibroblasts as observed by expression of the fluorescent actin probe F-tractin using live-cell spinning-disk confocal microscopy (Fig. 7, A and B; and Video 3). Expression levels of SNX9 are unchanged in OCRL-deficient cells compared with control RPE-1 cells (Fig. 7 C). However, immunolabeling for the endogenous protein revealed the accumulation of distinct SNX9 foci at the heads of the actin comets in OCRL knockout (KO) cells (Fig. 7, D and E; and Fig. S5 E), as previously observed in patient fibroblasts (Nández et al., 2014). Expression of a murine mCherry-OCRL rescue construct abolished the appearance of these comets (Fig. S5, F and G; and Video 4).

**Figure 8.**
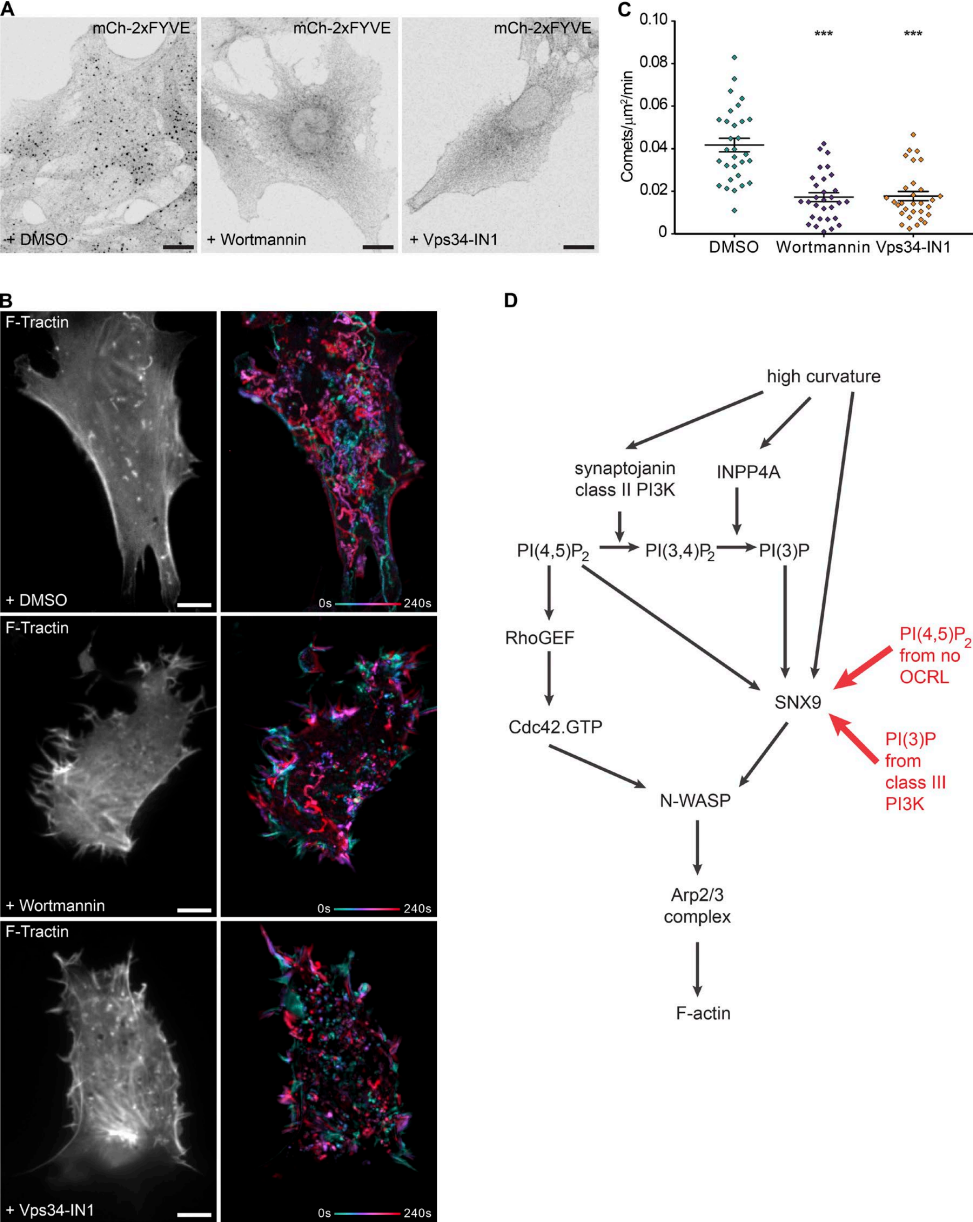
**PI 3-kinase inhibitors decrease the number of actin comet tails in OCRL-deficient cells and overall model.** (A) Purified mCherry-2×FYVE domain used to stain for PI(3)P in cells fixed after treatment with DMSO control, 2 µM wortmannin, or 10 µM Vps34-IN1 in serum-free media for 1 h before imaging. (B) Representative images from time-lapse videos of OCRL-deficient cells expressing F-tractin and treatment with DMSO or the inhibitors, as shown in Video 6. Actin comets were reduced on PI 3-kinase inhibitor treatment. Bars, 10 µm. (C) Quantification of actin comets from *n* = 30 cell regions for each treatment. Bars indicate the mean ± SEM for each condition. Difference assessed by ordinary one-way ANOVA with Dunnett’s multiple comparisons test, overall ANOVA, and comparing each inhibitor treatment to DMSO control cells. ***, P ≤ 0.0001. (D) Pathway of curvature signaling to actin polymerization via PI(4,5)P_2_/PI(3)P/SNX9 during CME. High curvature activates a cascade of phosphoinositol metabolism to change membrane identity and trigger actin polymerization at PI(4,5)P_2_/PI(3)P-enriched and highly curved sites via the oligomerization of SNX9 during endocytosis. In Lowe syndrome, OCRL deficiency increases PI(4,5)P_2_ levels on PI(3)P intermediates, triggering SNX9 assembly. PI(4,5)P_2_ also activates Cdc42 (independently of the curvature) for the relief of N-WASP inhibition. N-WASP oligomerization (via SNX9 binding) finally drives superactivation of the Arp2/3 complex and, thus, actin polymerization.

To reduce PI(3)P availability on the aberrant rocketing vesicles, we first targeted INPP4A, the endocytic source of PI(3)P we identified earlier. Treatment of OCRL KO cells with INPP4A siRNA resulted in a significant, ∼50% decrease in the number of actin comets (Fig. 7, F–H; and Video 5). There was no difference in the mean duration, distance, or speed of the comets tracked. To more broadly inhibit PI(3)P production, we treated OCRL-deficient cells with either a broad range or a more specific class III PI 3-kinase inhibitor. We used the irreversible covalent inhibitor wortmannin, which targeted PI(3)P production by class I (for example, via SHIP2 dephosphorylation of PI(3,4,5)P_3_), class III (that phosphorylate phosphatidylinositol on early endosomes), and class II PI 3-kinases (that are used during endocytosis to make PI(3,4)P_2_). To specifically inhibit class III PI 3-kinases, which are of particular interest because of the late CCP/early endosome character of the rocketing intermediates, we used Vps34-IN1 (Bago et al., 2014). Both wortmannin and Vps34-IN1 treatments caused a significant reduction in PI(3)P puncta in OCRL-deficient cells compared with controls, revealed by staining in fixed cells using the purified mCherry-2×FYVE domain (Fig. 8 A). Examination of actin comet tail formation in OCRL-deficient cells treated with the inhibitors revealed a >50% reduction in the number of actin comets for both wortmannin and Vps34-IN1 (Fig. 8, B and C; and Video 6). Though there was no change in the mean velocity of the tracked comets, there was a small but significant decrease in both mean track duration (14.48 ± 0.186 s control vs. 13.15 ± 0.04 s wortmannin [P = 0.0042] or 13.04 ± 0.2997 s Vps34-IN1 [P = 0.0027], overall ANOVA P = 0.0022) and distance traveled (2.26 ± 0.0461 µm control vs. 2.07 ± 0.04 µm wortmannin [P = 0.0404] or 1.995 ± 0.0594 µm Vps34-IN1 [P = 0.0099], overall ANOVA P = 0.0125). (Values are the means ± SEM, and statistics were assessed by ordinary one-way ANOVA with Dunnett’s multiple comparisons test). Thus, these data suggest that (a) PI(3)P is needed for actin comet tail formation, (b) the PI(3)P arises from INPP4A and class III PI 3-kinase routes of phosphoinositide conversion, and (c) PI 3-kinase inhibitors may provide a therapeutic option for disease symptoms.

## Discussion

We have discovered and fully reconstituted a SNX9-dependent signaling pathway that links its unique ability to coincidently detect PI(4,5)P_2_, PI(3)P, and membrane curvature with recruitment and activation of Cdc42, N-WASP, and Arp2/3 complex to trigger actin polymerization (Fig. 8 D). Given the well-established conversion of phosphatidylinositol that occurs during CME progression and the kinetic data on SNX9 (Taylor et al., 2011; Schöneberg et al., 2017), we propose that this pathway functions at late stages of CME, in concert with the curvature-dependent activity of the PI 4-phosphatase INPP4A to activate actin assembly at stalled CCP intermediates. SNX9 has recently been localized to highly curved regions of late CCPs by high precision using correlative single-molecule localization and EM, suggesting the coincidence of PI(4,5)P_2_ and PI(3)P at late budding profiles (Schöneberg et al., 2017; Sochacki et al., 2017).

Previous studies have suggested that PI(3,4)P_2_ functions to recruit SNX9 to late-stage CCPs (Posor et al., 2013; Schöneberg et al., 2017). However, our findings instead suggest that PI(3,4)P_2_ serves as a substrate for the INPP4A phosphatase to generate PI(3)P that functions synergistically with PI(4,5)P_2_ on the plasma membrane to recruit SNX9 and activate actin polymerization. Consistent with this, we show that INPP4A is necessary for SNX9 recruitment to CCPs.

We reconstituted SNX9-dependent actin polymerization from purified components to show that Cdc42 and N-WASP are both necessary and sufficient to activate the Arp2/3 complex in response to PI(4,5)P_2_/PI(3)P signals. Cdc42 activation responds to PI(4,5)P_2_ but not PI(3)P or membrane curvature. SNX9-mediated actin polymerization has previously been reconstituted on PI(4,5)P_2_ vesicles in a purified system similar to this one, but without Cdc42 (Yarar et al., 2007). We speculate that the difference in these results comes from our use of the N-WASP–WIP complex, which is more strongly autoinhibited than free N-WASP, as used previously (Yarar et al., 2007).

The SNX9 PX–BAR domain is uniquely structured to integrate all three combinatorial membrane signals. SNX9 binds to PI(4,5)P_2_-containing vesicles primarily via clusters of positive charges on the BAR domain and with lower affinity to PI(3)P-binding sites via the PX domain. Both sites are necessary for the high-affinity binding to PI(4,5)P_2_/PI(3)P-containing vesicles. In addition to specific lipid binding, we determined that SNX9 binds membranes of clathrin-coated vesicle–sized radius ∼10-fold better than those of lower curvature. Interestingly, the ability to bind two phosphoinositide lipids synergistically appears to be conserved and unique among the closely related SNX9/18/33 subfamily but not shared by other more distantly related PX–BAR domain–containing sorting nexins. The curvature dependence of INPP4A activity in generating PI(3)P works in concert with SNX9 to drive actin polymerization of curved PI(4,5)P_2_- and PI(3)P-containing membranes.

During maturation and the inward budding of CCPs, their increasing curvature activates phosphatidylinositol modifications via activation of synaptojanin (Chang-Ileto et al., 2011), class II PI 3-kinase (Hübner et al., 1998), and OCRL (Billcliff et al., 2016). We now show that this is also the case for a subsequent step, INPP4A hydrolysis of PI(3,4)P_2_ to PI(3)P. The production of PI(3)P acts as a second messenger for curvature through the multistep enzyme catalysis. As binding to PI(3)P by SNX9 is also curvature dependent, these combinatorial inputs trigger a signaling cascade toward actin polymerization (Fig. 8 D). To our knowledge, this work is the first demonstration of membrane curvature controlling a complex process (Arp2/3-mediated actin polymerization) downstream of an initial curvature-dependent event (INPP4A catalysis and SNX9 binding).

In CME, this signaling cascade could act as a timer: if vesicle scission occurs rapidly, PI(4,5)P_2_, PI(3)P, and curvature will not coincide and thus, CME will be actin independent. However, if scission is delayed (for example when membrane tension is high), the lifetime of this normally transient triple intermediate would be prolonged, allowing for SNX9 recruitment and the resulting actin polymerization to exert force on the membrane. The role of actin in CME has been debated but appears to be required at only a subset of CCPs (Taylor et al., 2011) or when membrane tension is high (Boulant et al., 2011). Consistent with this model, we observed a significant but modest inhibition of CME in HeLa cells treated with INPP4A that was no greater than the effect of disrupting actin assembly with latrunculin A. SNX9 knockdown has been similarly shown to have a modest effect on CME in most cells (Soulet et al., 2005; Bendris et al., 2016).

In agreement with our model, actin and SNX9 are co-opted when scission fails in dynamin KO cells (Ferguson et al., 2009). Likewise, in COS-7 cells, LifeAct fluorescence was observed during the final five frames of the clathrin signal at about half the budding CCPs, consistent with a model in which the triple PI(4,5)P_2_/PI(3)P/curved intermediate is only sometimes formed (Li et al., 2015). In yeast, where actin is used constitutively, there is no SNX9, and the molecular mechanism of actin involvement is different to the one we find here (Lewellyn et al., 2015). Though we have provided initial data that the pathway we identified biochemically is relevant to studies of CME, much further work is needed to fully understand how the pathway we have identified functions during CME. In view of our model, it will be particularly interesting to determine whether INPP4A and SNX9 are more strongly required in cells where actin is always used during CME (Grassart et al., 2014) or in high membrane tension conditions, such as in clathrin plaques where actin is used for scission (Boulant et al., 2011).

Previous observations had revealed that OCRL-deficient cells have aberrant actin organization and that reducing the levels of either N-WASP, SNX9, or PI 5-kinase, adding latrunculin B, or overexpression of the actin-severing protein cofilin all rescued actin organization and endosomal trafficking defects (Vicinanza et al., 2011; Nández et al., 2014). Here, we have shown that the normally transient coincidence of PI(4,5)P_2_ and PI(3)P is sustained in OCRL mutant cells and that the formation of actin comets on endocytic vesicles in OCRL mutant cells involves the SNX9 and INPP4A signaling cascade we have identified. By editing the genome of RPE-1 cells to mimic the OCRL deficiency in Lowe syndrome, we revealed that PI(3)P has a role in stimulating actin polymerization at the aberrant, still clathrin-coated (Nández et al., 2014) endocytic intermediates that rocket around these cells. siRNA knockdown and inhibitor studies established that not only is there a role for PI(3)P production via INPP4A, but also through PI 3-kinases, particularly class III PI 3-kinase. The effect of inhibiting PI(3)P production is substantial but not complete. This likely reflects the fact that actin polymerization can still be triggered by high PI(4,5)P_2_ observed in OCRL-deficient cells (Vicinanza et al., 2011), which can still recruit SNX9 and activated Cdc42. Nonetheless, these findings suggest the use of PI 3-kinase inhibitors initially developed for cancer indications as therapeutic approaches for Lowe syndrome (Rodon et al., 2013; Pasquier et al., 2015).

We demonstrate here how a trio of coincident effectors, PI(4,5)P_2_, PI(3)P, and membrane curvature, operates transiently to amplify and transduce signals from the membrane to locally activate the actin machinery. Our work provides a novel mechanism by which actin polymerization can be specifically triggered when and where it is needed in the cell. We show here that adapter proteins and the membrane environment can be critical factors driving actin polymerization at specific cellular locations. Arp2/3 complex–mediated actin nucleation is constitutively autoinhibited, and we demonstrate how a timed triple confluence of membrane signals unlocks an actin burst to power vesicle rocketing in health and disease.

## Materials and methods

This research has been regulated under the Animals (Scientific Procedures) Act 1986 Amendment Regulations 2012 after ethical review by the University of Cambridge Animal Welfare and Ethical Review Body.

### Plasmids

The all-in-one Cas9^D10A^ nickase vector was a gift from S. Jackson (Gurdon Institute, University of Cambridge, Cambridge, England, UK). Murine INPP4A variant 1 (muINPP4A1) cDNA ORF clone (GenBank accession no. NM_030266) was obtained from Origene, PCR amplified with Xho1 and SacII flanking restriction sites, and cloned into pmCherry-N1 to generate muINPP4A1-mCherry. Clathrin-Lca-mCherry was cloned from clathrin-Lca-EGFP (a gift from T. Kirchhausen, Harvard Medical School, Boston, MA). NeonGreen- and tomato F-tractin constructs were gifts from J.A. Hammer III (National Heart, Lung, and Blood Institute, National Institutes of Health, Bethesda, MD). Murine OCRL1-pmCherryC1 was a gift from C. Merrifield (Universite Paris-Saclay, Paris, France; plasmid 27675; Addgene; Taylor et al., 2011). *X. laevis* SNX9 was PCR amplified from pCMV-sport6-SNX9 (IMAGE clone 3402622; Source Bioscience) with FseI–AscI flanking restriction sites and cloned into pCS2-his-SNAP-FA to generate His-SNAP-SNX9. Similarly, *X. tropicalis* N-WASP (WASL) was PCR amplified from IMAGE clone 5379332 (Source Bioscience) and cloned into pCS2-his-SNAP-FA. Human N-WASP wGBD domain was PCR amplified from pCS2-mRFP-wGBD (a gift from W. Bement, University of Wisconsin-Madison, Madison, WI; plasmid 26733; Addgene) with XhoI–AscI flanking restriction sites and cloned into pET-pmKate2 to generate mKate-GBD. pCS2-His-Cdc42 (Q61L), pCS2-ZZ-TEV-WIP, pCS2-GFP-his-Cdc42, pGEX-NWASP-CA domain, and pGEX-NWASP-VCA domain plasmids were gifts from M. Kirschner (Harvard Medical School). Human Cdc42 was PCR amplified with FseI–AscI flanking restriction sites and cloned into pCS2-his-GST-FA to generate GST-Cdc42. *X. laevis* SNX9 mutants were designed based on sequence alignments with human SNX9 and mutants previously described for human SNX9 (Pylypenko et al., 2007). Site-directed mutagenesis of pCS2-his-SNAP-SNX9 was performed using the following primer pairs: Y276A/K302A (Y276A, 5′-GTAAACCACAGGGCTAAGCACTTTG-3′ and 5′-CAAAGTGCTTAGCCCTGTGGTTTAC-3′; K302A, 5′-CTCTTCCAGACGCGCAAGTTACAGG-3′ and 5′-CCTGTAACTTGCGCGTCTGGAAGAG-3′) and K511E/K517E (K511E, 5′-GTAAAGGAAAGTGATGAATTGGTGGTTGCAG-3′ and 5′-CTGCAACCACCAATTCATCACTTTCCTTTAC-3′; K517E, 5′-GGTTGCAGGTGAAATTACACAGC-3′ and 5′-GCTGTGTAATTTCACCTGCAACC-3′).

### Cell culture

Cell lines were incubated at 37°C in 5% CO_2_ and were passaged two to three times weekly. RPE-1 cells (a gift from S. Jackson, Gurdon Institute, University of Cambridge) were maintained in DMEM/F12 media supplemented with 10% fetal bovine serum, 0.25% sodium bicarbonate (Sigma-Aldrich), 100 µg/ml penicillin/100 U/ml streptomycin, and 2 mM l-glutamine (Gibco). Flp-In TRex HeLa cells (gift from S.S. Taylor, University of Manchester, Manchester, England, UK) were maintained in DMEM media (Gibco) supplemented with 10% fetal bovine serum, 100 µg/ml hygromycin B, and 5 µg/ml blasticidin S HCl (Invitrogen). To generate a stable inducible cell line, SNX9 was PCR amplified and cloned into a pcDNA5/FRT/TO vector modified to contain an N-terminal GFP tag. The construct was then cotransfected into cells with the Flp recombinase–encoding plasmid pOG44 as described by the vendor (Invitrogen). Recombinant GFP-SNX9 expression was induced by incubation with 1 ng/ml doxycycline hyclate (Sigma-Aldrich) for 24 h. For protein expression, Freestyle 293F cells (Thermo Fisher Scientific) were maintained in Freestyle 293 expression medium according to the manufacturer’s instructions.

### siRNA and plasmid transfection

siRNA sequences were transfected into Flp-In TRex HeLa cells using Lipofectamine 2000 (Invitrogen) according to the manufacturer’s instructions 72 h before analysis. The following siRNAs were used (all Stealth siRNA from Invitrogen): negative control (GC duplex medium), AP2M1 siRNA (AP2M1HSS101955), and INPP4A siRNAs (siRNA1, HSS105457; siRNA2, HSS105458; siRNA3, HSS105459). Constructs were transfected into Flp-In TRex HeLa cells using Lipofectamine 2000 24 h before analysis. In RPE-1 cells, Jetprime (Polyplus) was used to transfect in plasmid constructs 24 h before analysis, using a maximum of 2 µg DNA per 3-cm imaging dish. For siRNA treatment, cells were treated with two shots of siRNA at 48 and 24 h before analysis. The first transfection was performed using Lipofectamine RNAiMAX (Invitrogen) according to the manufacturer’s instructions, whereas the second was delivered 24 h before analysis concurrent with the DNA transfection using Jetprime, as outlined above. The following siRNAs were used: INPP4A siRNA1 (HSS105457; Invitrogen, as above) and siGENOME control nontargeting siRNA2 (D-001210-02-05; Dharmacon).

### CRISPR KO of OCRL in RPE-1 cells

The all-in-one Cas9^D10A^ nickase vector used was described previously (Chiang et al., 2016). Exon 9 of human OCRL was targeted using a single-guide RNA (sgRNA) pair designed using the Zhang laboratory CRISPR Design Nickase Analysis tool (Hsu et al., 2013). sgRNAs (sequences shown in Fig. S5) were synthesized with 5′-P-accg or 5′-P-aaac overhangs on the forward and reverse oligonucleotides, respectively, for restriction ligation (P, phosphate; Sigma-Aldrich). The annealed antisense and sense sgRNAs were sequentially cloned into the BsaI and BbsI restriction sites of the vector, respectively. RPE-1 cells were transfected with the plasmid (as above), and, after 24 h, transfected single cells transiently expressing RFP were individually deposited into 96-well plate wells by FACS to generate monoclonal cultures (Flow Cytometry Facility, Wellcome Trust–Medical Research Council Stem Cell Institute, University of Cambridge). Once individual clones had grown up, they were screened for OCRL expression by Western blot, with the sequence of positive clones lacking OCRL verified by PCR amplification of the genomic region around the exon 9 target site and with the bands representing each mutant allele extracted and sent for sequencing (Source Bioscience). The following primers were used for PCR amplification and sequencing of the product: forward, 5′-GCCCACTGCCTTTGTGTTCT-3′; reverse, 5′-TGTCACTGTTAGAACTGACCCCA-3′.

### Antibodies and fluorescent protein reagents

The following antibodies were used in this study: rabbit anti-Arp2 (sc-15389; [H-84]; Santa Cruz), rabbit anti-GST (ab9085; Abcam), rabbit anti-tRFP (AB234; Cambridge Bioscience), goat anti-INPP4A (sc-12314; [N-15]; Santa Cruz), rabbit anti-INPP4A (ab109622; [EP3425(2)]; Abcam), mouse anti-clathrin heavy chain antibody (610499; BD Transduction Laboratories), mouse anti-AP50/AP2M1 (611353; BD Transduction Laboratories), rabbit anti-SNX9 (a gift from S. Carlsson, Umeå University, Umeå, Sweden), mouse anti-SNX9 (ab118996; [2F1]; Abcam), rabbit anti-OCRL (ab181039; [EP10256]; Abcam), rabbit anti-RFP (600–401-379; Rockland), and mouse anti–α-tubulin (ab7291; [DM1A]; Abcam). Secondary antibodies were conjugated to Alexa Fluor 568 or 647 (Molecular Probes) for immunolabeling or to CW680 and 800 fluorescent probes (LI-COR Biosciences) for Western blotting. Pyrene actin was purchased from Cytoskeleton. Fluorescent actin was purchased from Thermo Fisher Scientific. 6×His-tagged human INPP4A was obtained from Echelon Biosciences.

### Immunoprecipitation, SDS-PAGE, and Western blotting

Cells were grown on plastic plates and were washed with ice-cold PBS. Ice-cold lysis buffer (20 mM Hepes, pH 7.4, 100 mM NaCl, 2 mM MgCl_2_, 0.1 mM EDTA, 1% NP-40, protease inhibitor cocktail [set III; Calbiochem], and phosphatase inhibitors, pH 7.4) was added to each plate and left on ice for 30 min before cells were scraped off, resuspended, and spun down at max speed in a tabletop centrifuge. To probe for INPP4A in Flp-In TRex HeLa cells, the supernatant was incubated with 10 µl of protein G magnetic beads (GE Healthcare) preincubated with 1 µg of goat anti-INPP4A overnight at 4°C. The beads were sedimented and washed extensively with lysis buffer and boiled in SDS sample buffer, and the eluted material was analyzed by Western blotting. For SDS-PAGE analysis, samples were boiled in SDS sample buffer, run on 4–20% polyacrylamide gels (BioRad) according to the manufacturer’s instructions, and analyzed by either adding InstantBlue (Expedeon) or by Western blotting using an iBlot 2 Dry Blotting System for transfer (Life Technologies), IRDye antigen detection, and an Odyssey Sa reader (LI-COR Biosciences).

### Protein purifications

Unless otherwise indicated, chemicals were purchased from Sigma-Aldrich. All steps were performed at 4°C, and purified proteins were concentrated using a 10,000 MWCO spin concentrator (Millipore). Their concentrations were determined based on their absorption at 280 nm, and they were stored at −80°C in 10% glycerol after snap freezing in liquid nitrogen. 

### Arp2/3 complex

Bovine Arp2/3 complex was purified as previously described, except using GST-CA rather than GST-VCA (Ho et al., 2006). Arp2/3 was purified from *X. laevis* egg extracts using a modified similar protocol. Crude cytosolic extract was prepared as described previously (Walrant et al., 2015), diluted 1:2 into MES buffer (100 mM MES, pH 6.8, 0.2 mM EGTA, 0.5 mM MgCl_2_, 0.1 mM EDTA, and 1 mM DTT), and centrifuged at 50,000 rpm for 1 h in a rotor (70Ti; Beckman Coulter). The supernatant was collected and dialyzed into SP buffer (50 mM MES, pH 6.8, 1 mM EDTA, 1 mM MgCl_2_, and 1 mM DTT) in a 10,000 MWCO cassette (Thermo Fisher Scientific) and then applied to a sulphopropyl fast flow (SP FF) column (Hi Trap; GE Healthcare) on an ÄKTA flow pressure liquid chromatography (FPLC) system (GE Healthcare). The column was washed with 10 mM KCl-SP buffer and then eluted with 80 mM KCl-SP buffer. Fractions containing Arp2/3 complex were pooled and dialyzed overnight into GST-CA loading buffer (20 mM Hepes, pH 7.4, 25 mM KCl, 1 mM MgCl_2_, 0.5 mM EDTA, 0.1 mM Na-ATP-Tris, and 1 mM DTT). A GST-CA column was prepared from BL21 pLysS *Escherichia coli* expressing the GST-CA construct. Bacteria were harvested, resuspended in buffer A (containing 150 mM NaCl, 20 mM Hepes, pH 7.4, 2 mM EDTA, and 2 mM DTT), and lysed by probe sonication. After ultracentrifugation (40,000 rpm for 45 min in a 70Ti rotor), the bacterial supernatant was incubated with glutathione–Sepharose 4B (GS) beads (GE Healthcare). After extensive washing in buffer A/high salt buffer A (300 mM NaCl), the dialyzed SP column elution was applied to the GST-CA column. After extensive washing in 0.2 M KCl-GST-CA loading buffer, samples were eluted in a disposable column by the addition of 0.5-ml aliquots of 0.2 M MgCl_2_-GST-CA loading buffer. Fractions containing Arp2/3 complex were pooled and underwent gel filtration on a gel filtration column (HiLoad 26/600 Superdex 200; GE Healthcare) into Arp2/3 storage buffer (10 mM Hepes, pH 7.4, 50 mM KCl, 1 mM MgCl_2_, 1 mM EGTA, and 1 mM DTT). 

### 6×His-SNAP-SNX9 and SNX9 mutants, 6×His-mKate-wGBD, 6×His-mCherry-2×FYVE, 6×His-eGFP-2×PH-TAPP1, and GST-SNAP-2×FYVE

pCS2 constructs were transfected into 293F cells by 293fectin reagent (Thermo Fisher Scientific) according to the manufacturer’s instructions 48 h before harvesting. 293F cell pellets were resuspended in buffer containing EDTA-free cOmplete protease inhibitor tablets (Roche) before lysis by probe sonication. pET and pGEX plasmids were transformed into BL21 pLysS *E. coli* and induced overnight at 19°C. Bacteria were harvested and lysed by probe sonication in buffers containing 150 mM NaCl and 20 mM Hepes, pH 7.4, with 2 mM 2-mercaptoethanol for 6×His tags and 2 mM EDTA and 2 mM DTT for the GST tag. After ultracentrifugation (40,000 rpm for 45 min in a 70Ti rotor), proteins were affinity purified on nickel–nitrilotriacetic acid (Ni-NTA) agarose beads (Qiagen) for the 6×His tag or GS beads (GE Healthcare) for the GST tag. GST proteins were cleaved using tobacco etch virus (TEV) protease. 6×His tag proteins were eluted from Ni-NTA beads by stepwise addition of increasing concentrations of 50–300 mM imidazole in a buffer also containing 20 mM Hepes, pH 7.4, 150 mM NaCl, and 2 mM 2-mercaptoethanol. Fractions containing cleaved or eluted proteins were pooled and purified further using S200 gel filtration on an ÄKTA FPLC. SNAP-SNXs in pGEX plasmids were transformed into BL21 pLysS *E. coli* and induced for 3 h at 37°C. Bacteria were harvested and lysed by probe sonication in buffers containing 150 mM NaCl and 20 mM Hepes, pH 7.4, with 2 mM DTT. After ultracentrifugation (40,000 rpm for 45 min in a 70Ti rotor), proteins were affinity purified on GS beads. GST proteins were cleaved using thrombin protease. Thrombin was finally inactivated by the addition of 1 mM PMSF. 

### Cdc42

For expression of GST-Cdc42, 293F cells were transfected as above, subcultured 48 h after transfection at a 1:2 ratio, and given a second transfection of the plasmid, with the cells harvested 24 h later. Isoprenylated GST-Cdc42 was purified from the membranes of 293F pellets and loaded with GTP-γS or GDP as described by Lebensohn et al. (2006), with the protein eluted in 50 mM Tris-HCl, pH 8.0, 10 mM reduced glutathione, 1 mM DTT, and 0.1% wt/vol sodium cholate hydrate according to the GS bead manufacturer’s instructions (GE Healthcare). Samples containing eluted protein were pooled and buffer exchanged by spin concentration into storage buffer. Isoprenylated hexahistidine-tagged Q61L Cdc42 was extracted from the membranes of 293F pellets as for GST-Cdc42 and purified on Ni-NTA agarose beads as above. 

### SNAP–N-WASP–ZZ-WIP

SNAP-tagged *X. tropicalis* N-WASP in complex with ZZ-tagged human WIP was purified from 293F cells based on methods previously described (Ho et al., 2004). The ZZ-WIP contains a TEV cleavage site between the tag and the protein. Lysates were incubated with IgG Sepharose 6 beads (GE Healthcare) in XB buffer (100 mM KCl, 0.1 mM CaCl_2_, 1 mM MgCl_2_, and 10 mM Hepes, pH 7.4). After extensive washing, TEV protease was added in XB buffer containing 10 mM DTT and 10% glycerol and incubated overnight to cleave the N-WASP–WIP complex from the beads. The disposable column was drained to collect the cleaved protein complex and applied to a small volume of GS beads to sequester the TEV protease. 

### Actin preparation procedure

Actin was prepared according to the previously published protocol (Pardee and Spudich, 1982). The muscle from the leg of a freshly killed rabbit (same day, kept on ice) was taken, removing fat and connective tissue. The muscle was minced twice with a meat grinder and extracted in Guba Straub buffer (0.3 M NaCl, 0.1 M NaH_2_PO_4_ [2H_2_0], 5 mM Na_2_HPO_4_, 1 mM NaN_3_, 0.05 mM PMSF, 1 mM MgCl_2_, and 1 mM Na_4_P_2_O_7_, pH to 6.5, adding 1 ml of 200 mM ATP just before use), stirring consistently for 10–15 min. The muscle was centrifuged at 3,000 rpm in a J6-MC centrifuge (rotor TY.JS 4.2; Beckman Coulter) precooled to 4°C. The muscle residue was resuspended in 1 liter of buffer containing 4% NaHCO_3_ and 1 mM CaCl_2_ plus 9 liters distilled H_2_O (dH_2_O), stirred for 15 min and filtered through four layers of cheesecloth. The muscle residue was diluted into 10 liters dH_2_O and quickly squeezed through cheesecloth (working quickly as muscle swells at low ionic strength, and F-actin is converted to G-actin and would be lost). The residue was suspended in 2.5 liters of cold acetone, stirred at room temperature for 15 min, and then filtered through cheesecloth. Acetone washing and filtering was repeated until the supernatant became clear (three to four times). The muscle powder was spread out on filter paper and dried in the fume hood overnight. Once dry, it was stored at −80°C. Stringy parts of the muscle acetone powder were separated, and 5 g was weighed out and added to 100 ml of ice-cold G-buffer (2 mM Tris, pH 8, 0.2 mM CaCl_2_, 0.5 mM NaN_3_, 0.2 mM Na_2_ATP, and 0.5 mM DTT). The mix was slowly stirred on ice for 30 min until viscosity increased. The suspension was carefully transferred into polycarbonate centrifuge tubes for the 70Ti rotor (Beckman Coulter), topped up with G-buffer, and centrifuged at 20,000 *g* for 35 min at 4°C. The supernatant was filtered through two small pinches of glass wool packed into the neck of a funnel and then filtered with a 0.45-µm filter, followed by 0.22-µm filters. At room temperature, a final concentration of 0.8 M KCl and 2 mM MgCl_2_ was added. The actin was stirred slowly and consistently for 30 min at room temperature to let it polymerize and then stirred gently at 4°C to dissociate contaminating tropomyosin. The suspension was poured into tubes for the rotor (70Ti) and centrifuged at 45,000 rpm (80,000 *g*) at 4°C to sediment the F-actin. 1–2 ml G-buffer was added, and the pellet was gently resuspended using a Teflon-coated rod. The actin was homogenized and transferred to a dialysis cassette. The actin was dialyzed in the cold room against 1 liter of G-buffer and then dialyzed again overnight. Dialysis continued by changing the G-buffer three times, each over the next 2 d in the cold room. On day 4, the dialysis buffer was changed two to three times over 4 h in the morning. Then, the actin was centrifuged at 80,000 *g* for 2 h to sediment aggregates. A gel filtration column (S200) was equilibrated with fresh G-buffer using an FPLC, and the G-actin was applied to the column and repeated as needed, avoiding the lower part of the tube containing the pellet of actin aggregates. The fractions were run on SDS-PAGE, and the pure actin-containing fractions were pooled and dialyzed into G-buffer minus NaN_3_. Sucrose was added to 5% final concentration, and 5-ml aliquots were snap frozen, dried in a freeze dryer, and stored at −80°C. Cold dH_2_O was added to resuspend the actin, which was then able to be stored on ice for up to 1 mo.

### Lipid protonation and liposome preparation

All lipids were purchased from Avanti Polar Lipids (natural brain lipids for PC, PS, and PI(4,5)P_2_, and synthetic for PI(3,4)P_2_ and PI(3)P), apart from C16-NBD–labeled PI(3,4)P_2_ [Echelon Biosciences], which was a gift from P. Vecino, Tebu-Bio). Phosphoinositide powders were resuspended in CHCl_3_, dried under a stream of nitrogen, and desiccated for 1 h. The lipid film was resuspended with 2 CHCl_3_/1 MeOH/0.001 1N HCl, incubated for 15 min, dried under nitrogen, and desiccated for 1 h. The lipid film was then resuspended with 3 CHCl_3_/1 MeOH, dried under nitrogen, resuspended with CHCl_3_, dried under nitrogen, and finally resuspended with CHCl_3_ at 1 mg/ml and stored at −20°C. Liposome samples for microscopy experiments (with a broad size distribution from 50 nm to 5 µm) were prepared at a 1-mM total lipid concentration by mixing lipid chloroform/methanol stocks as appropriate (with 0.2% rhodamine-PE or 0.6% NBD-PE) in a glass test tube and drying with nitrogen and then under vacuum for 1 h. Lipid films were resuspended in a sucrose solution with an osmolarity equivalent to XB buffer (204 mosM) and then shaken vigorously on a vortexer for 30 min at room temperature. Giant unilamellar vesicles were prepared from these samples by freeze/thawing in liquid nitrogen/37°C water bath five times (Traïkia et al., 2000), followed by gentle centrifugation to discard small liposomes and membrane residues. In parallel, large unilamellar vesicles and small unilamellar vesicles were prepared from the same sample by freeze/thawing as above eight times, followed by extrusion 15 times, initially through an 800-nm polycarbonate filter and then a 50-nm polycarbonate filter (Mini-Extruder; Avanti Polar Lipids). The giant, large, and small unilamellar vesicle samples were mixed together (molar ratio 3:1:1) and stored at 4°C before observation. SUV samples for sedimentation assays and microscopy assays were prepared at a 1-mM total lipid concentration by mixing lipid stocks as appropriate in a glass test tube and drying with nitrogen and then under vacuum for 1 h. Lipid films were resuspended in XB buffer for sedimentation assays or a sucrose solution with an osmolarity equivalent to XB buffer (204 mosM) for microscopy assays and then shaken vigorously on a vortexer for 30 min at room temperature. Liposomes were prepared by freeze/thawing in liquid nitrogen/37°C water bath eight times followed by extrusion 15 times initially through an 800-nm polycarbonate filter to prepare the largest liposomes and then a 100-nm polycarbonate filter. Loss of lipids during filtering was quantified using SDS-PAGE and Coomassie staining, after which the lipid concentration of the 100-nm vesicles was adjusted to match the 800-nm sample.

### Liposome size and surface area determination by PAINT and analysis

Superresolution imaging was performed using a home-built TIRF mode based on an inverted optical microscope (IX73; Olympus) and coupled to an electron-multiplied charged-coupled device (EMCCD) camera (Evolve II 512; Photometrics). A continuous wave diode-pumped solid-state laser operating at 532 nm (LASOS; Lasertechnik GmbH) was used as the excitation light source to induce fluorescence in the Nile red molecules. The laser was prepared to circularly polarized light by a quarter wave plate and directed off a dichroic mirror Di02-R532-25 × 36 for 532 nm of illumination (Semrock) through a high numerical aperture oil-immersion objective lens (Plan Apochromat 60× NA 1.49; APON 60× OTIRF; Olympus) to the sample coverslip. Total internal reflection was achieved by focusing the laser at the back focal plane of the objective, off axis, such that the emergent beam at the sample interface was near collimated and incident at an angle greater than the critical angle θ_c_ ∼67**°** for a glass/water interface for TIRF imaging. This generated an ∼50-µm diameter excitation footprint with power densities in the range of ∼0.5 kWcm^−2^ at the coverslip. The emitted fluorescence was collected through the same objective and further filtered using a longpass filter (BLP01-532R-25; Semrock), a bandpass filter (FF01-650/200-25; Semrock), and a shortpass filter (FF01-715/SP-25; Semrock) for 532 nm of illumination before being expanded by a 2.5× relay lens (PE 2.5× 125; Olympus). The fluorescence image was captured on the EMCCD camera running in frame transfer mode at 20 Hz, with an electron multiplication gain of 250, operating at −70°C with a pixel size of 16 µm and automated using the open source microscopy platform Micromanager. Nile red (N1142; Thermo Fisher Scientific) stock solutions were prepared by dissolving Nile red into DMSO (D2650 >99.7% purity) to a concentration of 1 mM. The stock solution was divided into 10-µl aliquots, flash frozen in liquid N_2_, and stored in the dark at −80°C until required. For a working solution, the aliquot was diluted into the filtered XB buffer to a final concentration of 50 nM. For the observation chamber, glass coverslips (0.13-mm thickness, 22 × 22 mm, VWR collection, 631–0124) were cleaned by using an argon plasma cleaner (PDC-002; Harrick Plasma) for at least 1 h. After that, frame-seal slide chambers (9 × 9 mm; BioRad) were firmly affixed to the cleaned glass coverslips. The chamber was covered with poly-l-lysine solution (0.01% wt/vol, P4832-50ML) to fully coat the coverglass surface, incubated for 30 min, and then washed at least four times with the filtered XB buffer. After that, the chamber was covered by the filtered XB buffer to prevent any contaminations of the coverslip’s surface until loaded with the liposome samples. To determine the liposome size, 500 nM (100-nm liposomes) and 1 µM (250-nm liposomes) concentrations were added to the chamber and incubated for 10 min, and then 1 µl of the working Nile red solution (50 nM) was added to the chamber to a final concentration of 1 nM. Superresolution images of 100-nm and 250-nm liposomes on a glass surface were observed during 2,000 frames at a frame rate of 20 ms. Nile red localizations in the image were determined using the Peak Fit plugin for ImageJ (National Institutes of Health). Individual liposome diameters in the superresolution image were determined by calculating the full width half maximum (FWHM) from the standard deviation of a two-dimensional Gaussian fit. With the FWHM value, we calculated the surface area (A) of individual liposomes using the equation 

$$A=\;4{\mathrm{\pi r}}^{2}\;\approx \;4\pi {\left(\frac{\mathrm{FWHM}}{2}\right)}^{2}.$$

### Liposome size determination by dynamic light scattering

Liposome samples were measured by dynamic light scattering using a molecular size analyzer (Zetasizer Nano ZS; Malvern Instruments), with liposomes at a 150-mM final concentration in XB buffer. The size distribution output in intensity was converted into number distribution using the Zetasizer software transformation method, with the following parameters: shell refractive index at 1.43, particle core refractive index at 1.33, and core radius/shell ratio at 20 for 400-nm extruded liposomes and 10 for 100-nm extruded liposomes. Samples were stored on ice before sedimentation assays.

### Liposome in vitro imaging and analysis

The observation chamber was prepared by adding a 50-µl drop of 5% BSA solution (wt/vol, in water, filter with 0.22-µm membranes) to a silicon gasket on a coverslip and incubating for 1 h, after which the area was extensively rinsed with XB buffer. To test for SNX9 binding, 45 µl of XB was applied to the chamber, followed by 3 µl liposome mix (rhodamine-PE labeled), which was left for 5–10 min to sediment, after which 125 nM (final) Alexa Fluor 647–SNAP-SNX9 was slowly injected on top of the sample, and several representative regions were imaged (immediately after injection). To test for actin polymerization in the presence of frog egg extract, 22 µl XB was applied to the chamber, followed by 3 µl liposome mix, which was left to sediment for 5–10 min. 35 µl of reaction mix was prepared in XB containing (final assay concentration after addition to liposome mix) energy mix (5.25 mM phosphocreatine, 0.7 mM ATP, 0.7 mM MgCl_2_, pH 7, 2 mM DTT, 3.5 mg/ml HSS frog egg extract, 0.8 mM unlabeled actin, 0.3 mM Alexa Fluor 647–labeled actin, and ±100 nM SNX9). These were added to the sample, and several representative regions were imaged after 10 min of incubation. To test the activity of the phosphatase INPP4A, the same protocol was used, except that liposome samples were preincubated with the enzyme before sedimentation (20 nM final; 1 h at 25°C in the dark). To examine actin polymerization in the presence of the minimal purified system, 21 µl XB was applied to the chamber, followed by 3 µl liposome mix, which was left to sediment for 5–10 min, after which a final assay concentration of 50 nM GTP-γS–loaded Cdc42 was gently added on top of the sample. 25-µl reaction mixes were prepared in XB containing (final assay concentration) 0.2 mM ATP, 2 mM DTT, 8 µM unlabeled actin, 0.3 µM Alexa Fluor 647–labeled actin, 50 nM *X. laevis* Arp2/3 complex, 100 nM SNX9, and 100 nM N-WASP–WIP and were added to the sample, and several representative regions were imaged after 10 min of incubation. Images were analyzed using MetaMorph software.

### Sedimentation assays

For SNX9 (WT and mutants), SNX2, SNX4, SNX5, SNX8, SNX18, and SNX33, 20 µl liposomes (1 mM total lipid) was mixed with proteins (1.5 µM final) in a 100-µl final volume of XB. These were incubated for 20 min at room temperature, followed by ultracentrifugation at 120,000 *g* (TLA-100 rotor; Beckman Coulter). For Cdc42 activation, 25 µl liposomes (1 mM) were mixed with HSS (3.5 mg/ml final) and mKate GBD (10 nM final). These were incubated for 15 min at room temperature, followed by ultracentrifugation at 120,000 *g*. Supernatants and pellets (resuspended in 100 µl XB) were collected and analyzed by SDS-PAGE Coomassie staining (SNX9) or Western blotting (mKate-GBD). Quantification was performed using the LI-COR imaging system and Image Studio software.

### INPP4A phosphatase thin-layer chromatography (TLC) assay

Silica TLC plates (Merck Millipore) were preimpregnated in a solution of 2 mM EDTA and 1% potassium oxalate/MeOH (2:3). 48% PC/47.5% PS/4% PI(3,4)P_2_/0.5% C16-NBD PI(3,4)P_2_ liposomes were prepared to a 2-mM total lipid concentration and extruded through 100-nm or 800-nm polycarbonate filters. The 800-nm extruded population had a mean diameter of 250 nm. 110 µl of each liposome suspension was incubated with 10 nM INPP4A at room temperature in the dark in a buffer containing 150 mM NaCl, 20 mM Hepes, pH 7.4, 2 mM EDTA, and 5 mM CaCl_2_. 10-µl samples were taken after 0, 1, 2, 5, 10, 20, 30, 60, 90, 120, and 150 min and immediately boiled to inactivate the enzyme. For each time point sample, lipids were extracted by adding 10 µl chloroform, shaking vigorously, and the organic phase was collected. The extraction was repeated, and the organic phases were pooled, with 10 µl lipid solution in chloroform for each time point spotted on a TLC plate. The plate was eluted with 65% propan-1-ol/35% 2 M acetic acid for several hours in the dark, after which the plates were read on a phosphorimager (Fujifilm) with the band intensity analyzed using the gel tool in ImageJ. Graphs were made in Excel (Microsoft).

### Pyrene actin assays

Pyrene actin assays were performed using the purified minimal reconstituted system based on a previous protocol (Ho et al., 2006). Actin was purified as previously described (Pardee and Spudich, 1982). 100-µl assay mixes were prepared in a quartz cuvette by adding components in the following order: 10× assay buffer (500 mM KCl, 100 mM Hepes, pH 7.4, 20 mM MgCl_2_, 0.2 mM ATP, and 2 mM DTT),100 µM (total lipid concentration) liposomes, 500 nM Q61L Cdc42, 100 nM SNX9, 100 nM N-WASP–WIP, and 50 nM bovine Arp2/3 complex, with reactions started by the addition of a 67% unlabeled/33% pyrene actin mix at a final actin concentration of 1.2 µM. For each series of experiments, a negative control of background actin polymerization in assay buffer and a positive control of using GST-VCA to activate the Arp2/3 complex were performed. Polymerization curves were collected on a spectrophotometer (FluoroMax-4; Horiba Scientific) and analyzed in Excel.

### Transmission EM

Samples were prepared as for the liposome minimal purified system microscopy assay and verified for actin aster formation by fluorescence microscopy immediately before negative staining. Small drops of samples were dotted onto dental film and glow discharged, and formvar-carbon–coated EM grids (provided by the Cambridge Advanced Imaging Centre, University of Cambridge) were inverted onto the samples for 2 min, after which they were quickly spotted on filter paper and immediately transferred to a spot of 3% aqueous uranyl acetate for 1 min. Excess negative stain was removed with filter paper, and the grids were allowed to dry before imaging on a transmission electron microscope (Tecnai G2; 80–200 kv). Images were cropped in Photoshop (Adobe).

### Fluorescence microscopy

For liposome in vitro analysis, confocal images were acquired on an inverted microscope (Ti-E; Nikon) equipped with spinning-disk (X-light Nipkow; Crest), 250-µm piezo-driven Z stage/controller (NanoScanZ) and Lumencor Spectra X LED illumination using a 100× 1.40 oil objective (Plan Apo VC; Nikon). Images were collected at room temperature with an EMCCD (Evolve Delta; Photometrics) camera in 16-bit depth using Metamorph software version 7.8.2.0. Alexa Fluor 488, 568, and 647 samples were visualized using 470/40, 560/25, and 628/40 excitation and 525/50, 585/50, and 700/75 emission filters, respectively. Live-cell imaging of actin comets in RPE-1 cells was performed on the same microscope supplemented with a stage top heated chamber (Okolabs) at 37°C. Cells were grown in glass-bottomed dishes (Ibidi), transfected as outlined above, and imaged in phenol red–free media containing 20 mM Hepes, pH 7.4. Confocal imaging of immunolabeled cells was performed on a laser-scanning microscope (FV1000; Olympus) using a 100× UPlanSApo 1.4 oil objective controlled by FV10-ASW software version 4.2. Alexa Fluor 488, 568, and 647 samples were imaged at room temperature using laser excitation lines at 488, 561, or 635 nm and the standard FV1000 photomultiplier tube detectors with emission windows set to 500–545 nm, 570–625 nm, and 655–755 nm, respectively. For live-cell imaging of CME in HeLa cells, cells were grown and transfected according to standard protocols on uncoated MatTek dishes. Experiments were conducted using a growth chamber (37°C, 5% CO_2_) in connection with an A1 R laser-scanning confocal microscope system with an EMCCD camera (ANDOR iXon; Nikon Instruments) under control of the NIS-Elements microscope imaging software, allowing real-time TIRF acquisitions with a 100× lens (Apochromat 1.49 oil 0.13–0.20 DIC N2; Nikon).

### Analysis of CME in HeLa cells

The representative microscopic images viewed in the figures were drift corrected using ImageJ and prepared (cropped, rotated and linearly adjusted for intensity) using Photoshop CS5. Spots of GFP-SNX9 or clathrin-Lca-mCherry after siRNA transfection were evaluated by analyzing TIRF live-cell acquisitions (800 ms between frames, over 193 s) using the Imaris software V7.5 (Bitplane), tracking mode. A region of interest sized 100 × 100 pixels (256 µm^2^) was segmented for spots using an estimated diameter of 0.4 µm, background subtraction, a filter using a quality threshold >102, Brownian motion, maximum distance of 0.7 µm, maximum gap size of 1, no fill gap, and a track duration minimum of 32 s. Graphs were made in Excel and Prism (GraphPad). Analysis of persistent CCPs and endocytic lifetimes was performed in MatLab (MathWorks) using custom-written software (Aguet et al., 2013). The latest software is available for download from M. Mettlen. In brief, the analysis focuses on diffraction-limited objects, and the detection of all clathrin-labeled structures is based on the assumption that all relevant fluorescent signals can be described by a Gaussian point spread function. Signals were selected as valid detections if their amplitude was higher than a 95th percentile confidence threshold in the local background noise distribution. Fluorescent trajectories were automatically calculated from the detections obtained in individual frames, and bona fide CCPs that undergo stabilization and maturation were distinguished from transient subthreshold clathrin-labeled structures based on quantitative analysis of the progression of their fluorescence intensity during early stages of growth, as previously described (Aguet et al., 2013). The lifetimes (i.e., time elapsed between first and last detection of a given trajectory) of these bona fide CCPs were then extracted and plotted. Tracks were categorized as persistent if their diffraction-limited fluorescent signal was present throughout the entire acquisition period (i.e., present in the first and last frames of the time lapse).

### Transferrin internalization assay

Flp-In TRex GFP-SNX9 HeLa cells were treated with 60 nM stealth siRNA (Invitrogen) for 72 h before uptake. The day before the assay, 10^5^ cells/well were seeded on coverslips in 24-well plates and induced with 1 ng/ml doxycycline (Sigma-Aldrich). Cells were pretreated with 1 µM latrunculin A (Sigma-Aldrich) or DMSO in medium for 10 min at 37°C immediately before addition of transferrin. Medium with 8 µg/ml 650-s-s-transferrin together with DMSO or 1 µM latrunculin A was then added to each well. After a 10-min incubation at 37°C, the medium was removed and cells were quickly washed once with PBS followed by cell fixation for 20 min with 3% PFA in PBS at room temperature. Cells were then washed once in 4°C stripping buffer (100 mM NaCl, 50 mM Tris-HCl, and 2.5 mM CaCl_2_, pH 8.7) and incubated two times for 20 min with freshly prepared 100 mM Mesna (Sigma-Aldrich) in stripping buffer. Cells were then incubated with 1:25,000 DAPI in PBS for 15 min and mounted for microscopy. Z stacks of the samples were imaged with a spinning-disk confocal live-cell imaging system (Cell Observer; Zeiss), controlled by ZEN interface with an inverted microscope (Observer.Z1; Axio) and equipped with a spinning-disk unit (CSU-X1A 5000) and an EMCCD camera (iXon Ultra; ANDOR). Endosomes were segmented as spots using IMARIS. Total intensity of detected endosomes in the Z stacks was divided by the amount of cells in the image (DAPI). Data were prepared, analyzed, and presented in Excel and Prism.

### Tracking analysis of actin comets in RPE-1 OCRL KO cells

Images were captured as a small Z series (4 × 0.2 µm), taken every 2 s over a 4-min period. Representative images and videos show a maximal projection of this Z stack and were prepared in FIJI (ImageJ). FIJI was also used to create the temporally color-coded images; the representative videos were bleach corrected, and a maximal projection of the Z stack was created. Background subtraction was performed by creating a mean intensity projection of the entire time course and subtracting this from each individual frame. The resulting stack was fed into the Temporal Color-Code plug-in using the ice LUT to create the images presented.

Comet tracking analysis was performed using the Trackmate plug-in in FIJI. Images were blinded before analysis and then processed to isolate moving comets and allow the plug-in to detect them. Each image was converted to a Z series maximal projection. The triangle threshold function was used to identify cells in the image, followed by an erosion function to remove outer processes (such as filopodia or membrane ruffling, which the plug-in could detect as motile puncta and attempt to track). This region was isolated, and the area of interest was recorded. After this, a stack of the difference between adjacent frames in this isolated region was created to isolate objects that had moved. This was analyzed using the Trackmate plug-in. Images were analyzed using the LoG detector with the median filter and subpixel localization turned on. Spots representing comet heads were detected using an estimated blob diameter of 0.75 µm, and an appropriate threshold was set manually. Identified blobs were determined as comets by tracking their movement using the Simple LAP tracker, using a maximum linking distance of 2.4 µm (determined by manually tracking a selection of control comets from several reference cells), with no gap closing. Detected tracks were categorized as comets when they persisted for 8 s or longer (five consecutive frames), with the number detected for each region recorded. The duration, track displacement, and mean track speed for all the comets in the image were also recorded. For data analysis, each cell region represents the number of comets detected in an individual image. These normally come from a single cell, but can include more than one cell when neighboring cells are also expressing F-tractin and therefore included after the thresholding step, with the data expressed as the number of tracks detected/thresholded area (micrometers squared)/total time (minutes). Graphs and statistical analysis were prepared using Prism software.

### Immunolabeling of RPE-1 OCRL KO cells

Cells grown on coverslips were fixed based on the Golgi fixation protocol outlined by Hammond et al. (2009). Washes were performed between each step. Cells were fixed in 2% PFA (Thermo Fisher Scientific) in PBS for 15 min and permeabilized in 20 µM digitonin (Sigma-Aldrich) in buffer A (150 mM NaCl, 20 mM Hepes, pH 7.4, and 2 mM EDTA) for 5 min. Cells were blocked in 5% goat serum (in buffer A; Sigma-Aldrich) for 45 min and then treated with primary antibody for 1 h (in 0.5% goat serum in buffer A), followed by secondary antibody for 30 min, before being postfixed in 2% PFA for 5 min. Coverslips were mounted onto slides using Hydromount (National Diagnostics). To label PI(3)P, purified mCherry-2×FYVE was added during the blocking step at a final concentration of 1 µg/ml and then immunolabeled using an RFP primary antibody and Alexa Fluor 568 secondary antibody. Images were captured as a laser-scanning confocal Z series (10 × 0.2 µm) and are displayed as a maximal projection of this stack, prepared in FIJI.

### OCRL actin comet inhibitor treatments

Wortmannin (Sigma-Aldrich) and Vps34-IN1 (Selleck Chemicals) were made up in DMSO at 2- and 10-mM stock concentrations, respectively. Cells were treated with wortmannin at a final concentration of 2 µM, Vps34-IN1 at 10 µM, and DMSO at an appropriate 1:1,000 dilution in serum-free media 1 h before analysis.

### Online supplemental material

Fig. S1 shows the SDS-PAGE of protein purifications for the reconstitution. Fig. S2 shows Western blots and gels of the SNX9 binding data and control showing sedimentation, and floatation assays give similar data. Fig. S3 has example pictures and the quantifications of liposome size from PAINT and dynamic light-scattering experiments. Fig. S4 shows control experiments for the effect of INPP4A depletion on endocytosis in HeLa cells. Fig. S5 illustrates how the OCRL CRISPR KO cells were made and control experiments for analysis of actin comet tails in these cells. Videos 1 and 2 show SNX9 and clathrin spots in control and INPP4A siRNA–treated HeLa cells. Videos 3 and 4 show actin comet tails in control, OCRL KO, and rescue RPE cells. Videos 5 and 6 show actin comet tails on INPP4A siRNA or inhibitor treatment.

## Supplementary Material

Supplemental Materials (PDF)

Video 1

Video 2

Video 3

Video 4

Video 5

Video 6
