# Supplementary material for: Control of actin polymerization via the coincidence of phosphoinositides and high membrane curvature
Source: J Cell Biol. 2017 Nov 6;216(11):3745–65. doi: 10.1083/jcb.201704061 (PMC5674896; doi:10.1083/jcb.201704061)
Supplement: Supplemental Materials (PDF) [file JCB_201704061_sm.pdf]

Daste et al., https://doi.org/10.1083/jcb.201704061

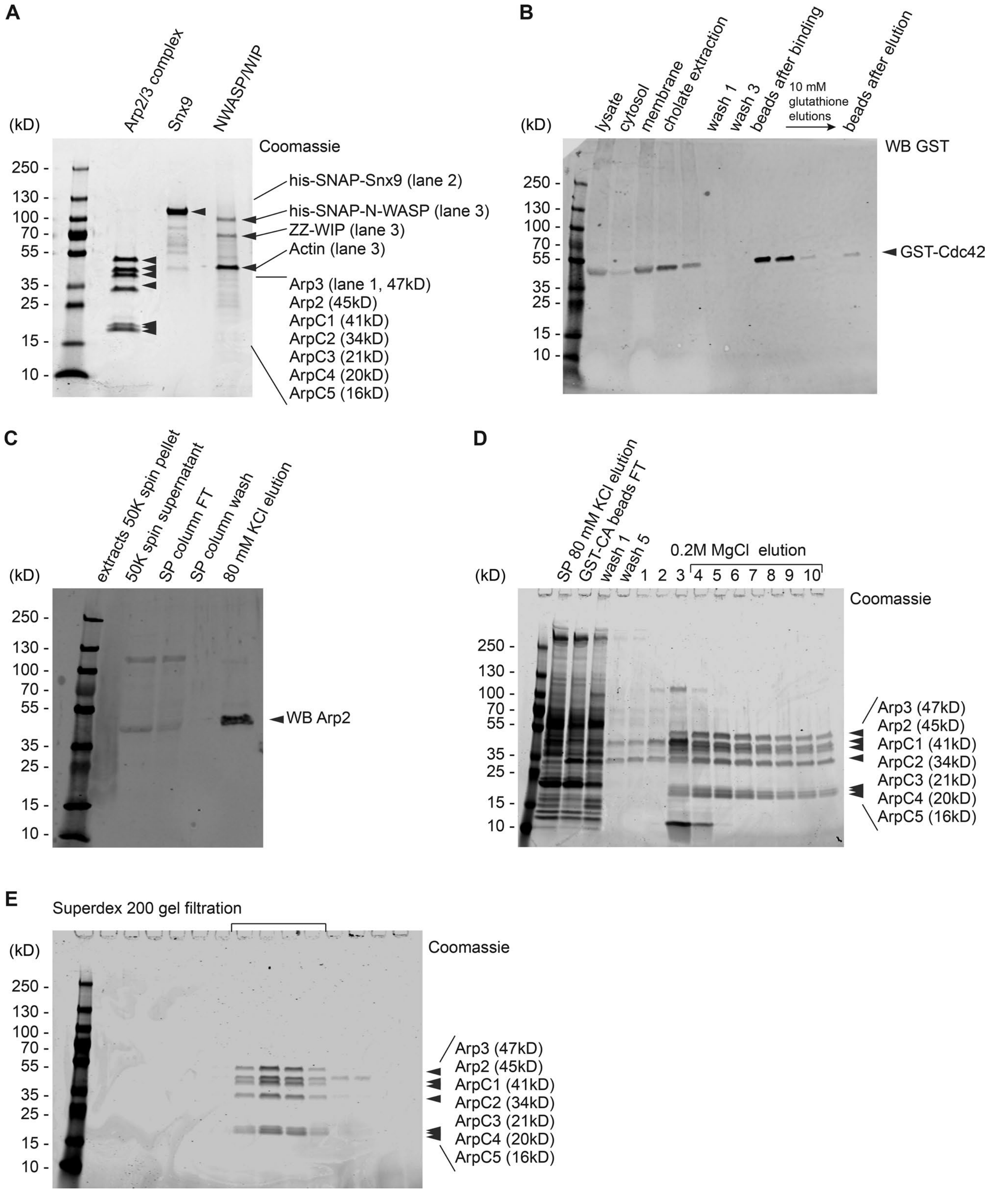

Figure S1. **Minimal system protein purifications.** (A) SDS-PAGE and Coomassie staining of purified Arp2/3 complex, SNX9, and N-WASP–WIP as used in the minimal reconstitution system (Fig. 2). (B) SDS-PAGE and Western blot (WB) of prenylated Cdc42 membrane extraction and purification. (C–E) Detailed purification of Arp2/3 complex from *Xenopus* egg extracts: Western blot against Arp2 for samples from the SP column step (C), SDS-PAGE and Coomassie staining of purification from the GST-CA column (D), and SDS-PAGE and Coomassie staining of the output after Superdex 200 gel filtration (E).

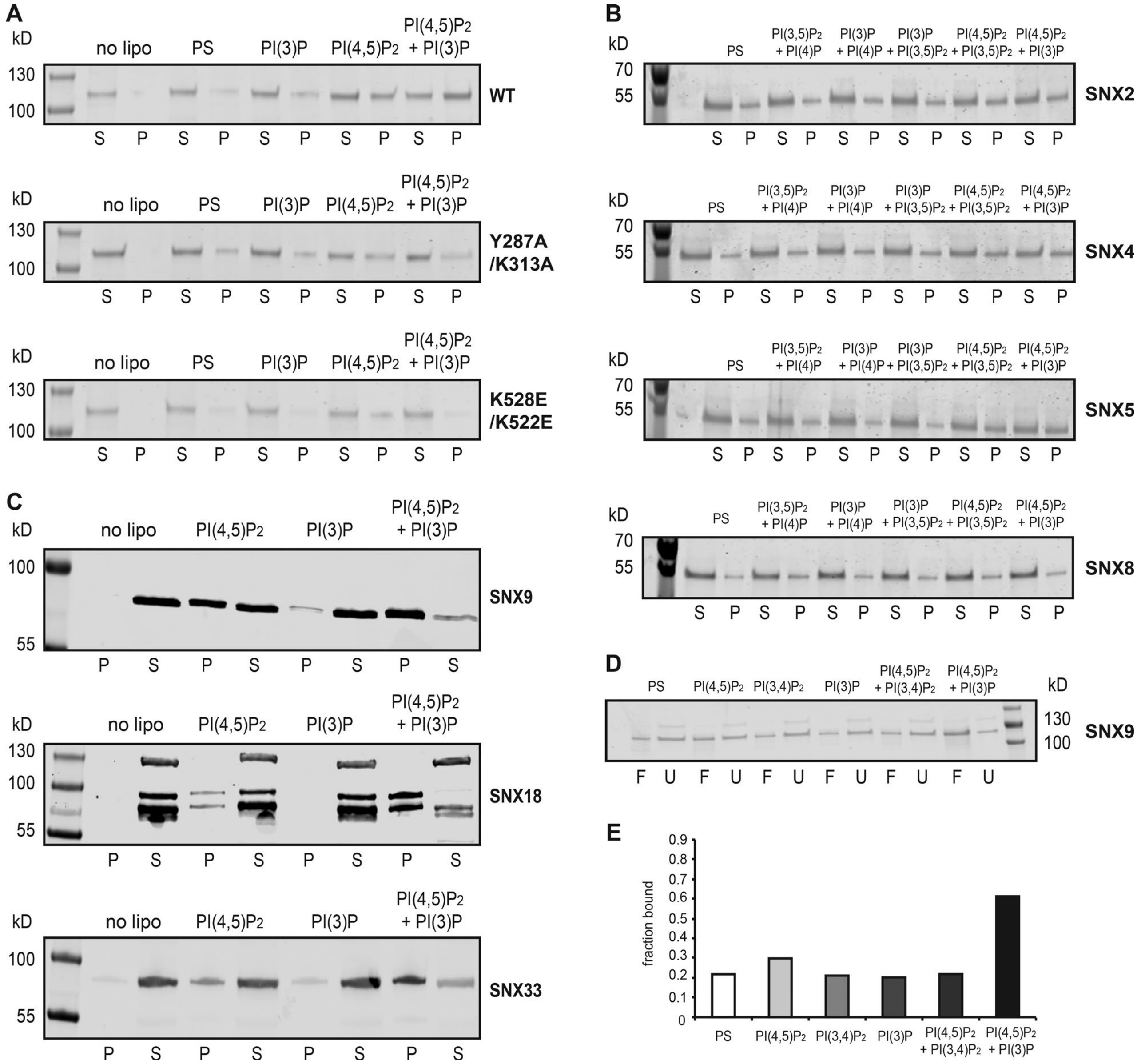

Figure S2. **Binding characteristics of SNX family proteins to different phosphoinositide combinations and examination of the curvature sensitivity of Cdc42 activation.** (A) Representative Coomassie-stained gels of sedimentation assays assessing the ability of WT or mutant SNX9 to bind PI(4,5)P_2, PI(3)P individually, or when combined together. PS indicates a control composition containing 70% PC/30% PS. S, supernatant; P, pellet. Example blot for the analysis shown in Fig. 3 B. (B) Representative Coomassie-stained gels of sedimentation assays assessing the ability of SNX2, 4, 5, and 8 to bind various phosphoinositide compositions. Example blot for the analysis shown in Fig. 3 D. (C) Representative Western blots of sedimentation assays using HSS assessing the ability of SNX9, 18, and 33 to bind PI(4,5)P_2 or PI(3)P individually or when combined together. Example blot for the analysis shown in Fig. 3 E. (D) Representative Western blots of flotation assay assessing the ability of SNX9 in various phosphoinositide compositions. F, floated; U, unfloated. (E) Quantification of the flotation assay, indicating the same specificity for the combination of PI(4,5)P_2 + PI(3)P seen in sedimentation assays is maintained. Representative data of two independent experiments.

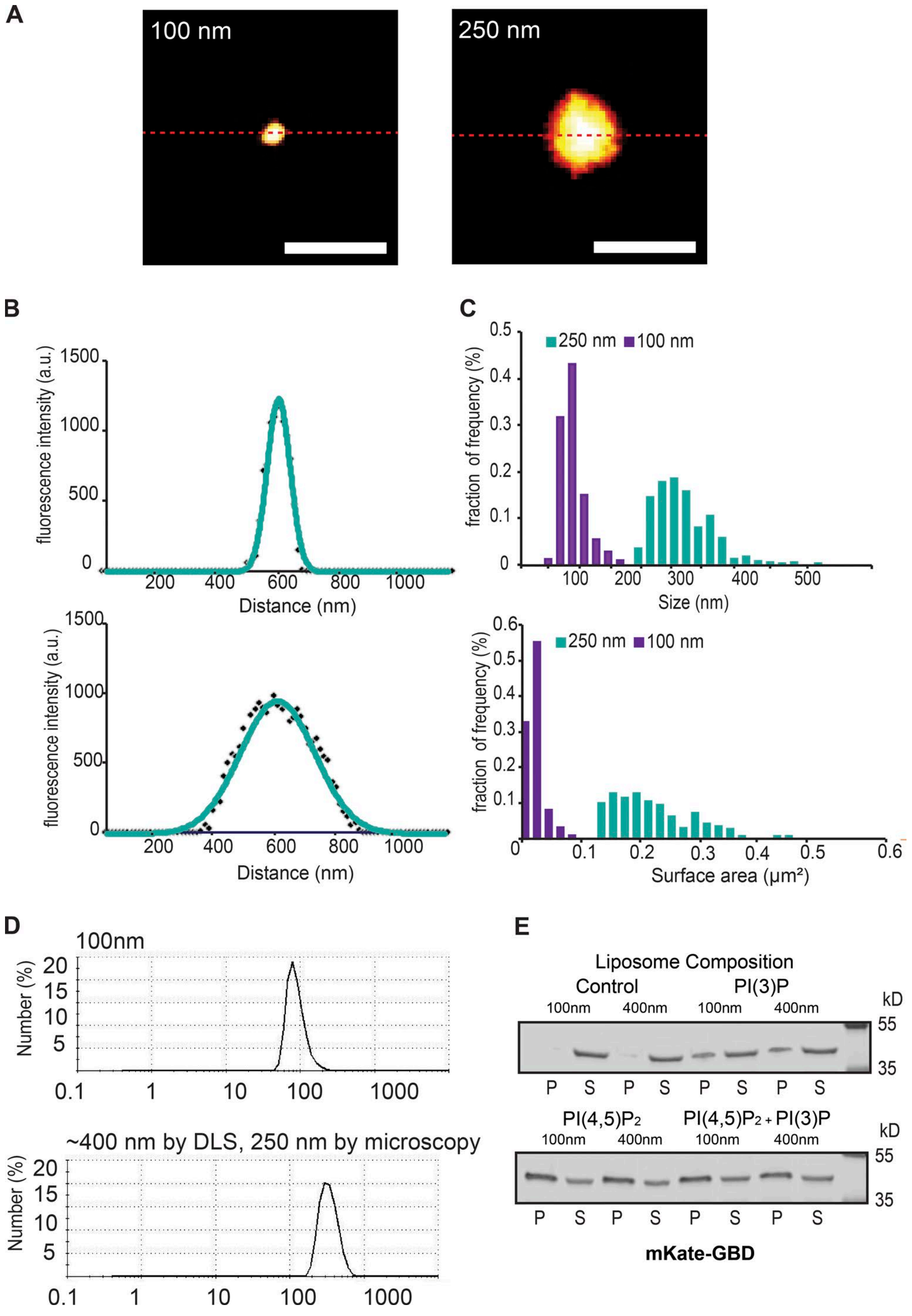

Figure S3. **Measurement of liposome sizes and surface area distributions.** (A) Representative superresolved PAINT images of 100- and 250-nm liposomes containing 4% PI(4,5)P_2, 1% PI(3)P, 65% PC, and 30% PS using Nile red. Bars, 500 nm. (B) The Gaussian fitting line gives the sigma (σ) and FWHM values (100-nm preparation: σ = 40.9 nm; FWHM = 96.2 nm; 250-nm preparation: σ = 133.8 nm, FWHM = 315.2 nm). (C) Histograms of both the size and surface area of 100- and 250-nm liposomes preparations. The histograms of the diameter of each liposome were calculated from the FWHM of the standard deviation of the two-dimensional Gaussian fit. 100-nm liposome: n = 755. 250-nm liposome: n = 401. The mean sizes are 91 nm and 265 nm for 100- and 250-nm liposomes, respectively. Histograms of surface area of individual liposomes were determined from FWHM of the standard deviation of the two-dimensional Gaussian fit. The mean values of the surface area are 0.03 μm^2 and 0.23 μm^2 for 100- and 250-nm liposomes, respectively. (D) Dynamic light-scattering (DLS) measurements assessing the diameter of liposomes used in Fig. 4. Data in B and D are representative of two independent experiments. (E) Representative Western blots of sedimentation assays assessing the ability of mKate-GBD to bind liposomes of distinct sizes for various phosphoinositide compositions, indicating the activity of Cdc42·GTP. Example blot for the analysis shown in Fig. 4 E. a.u., arbitrary units. S, supernatant; P, pellet.

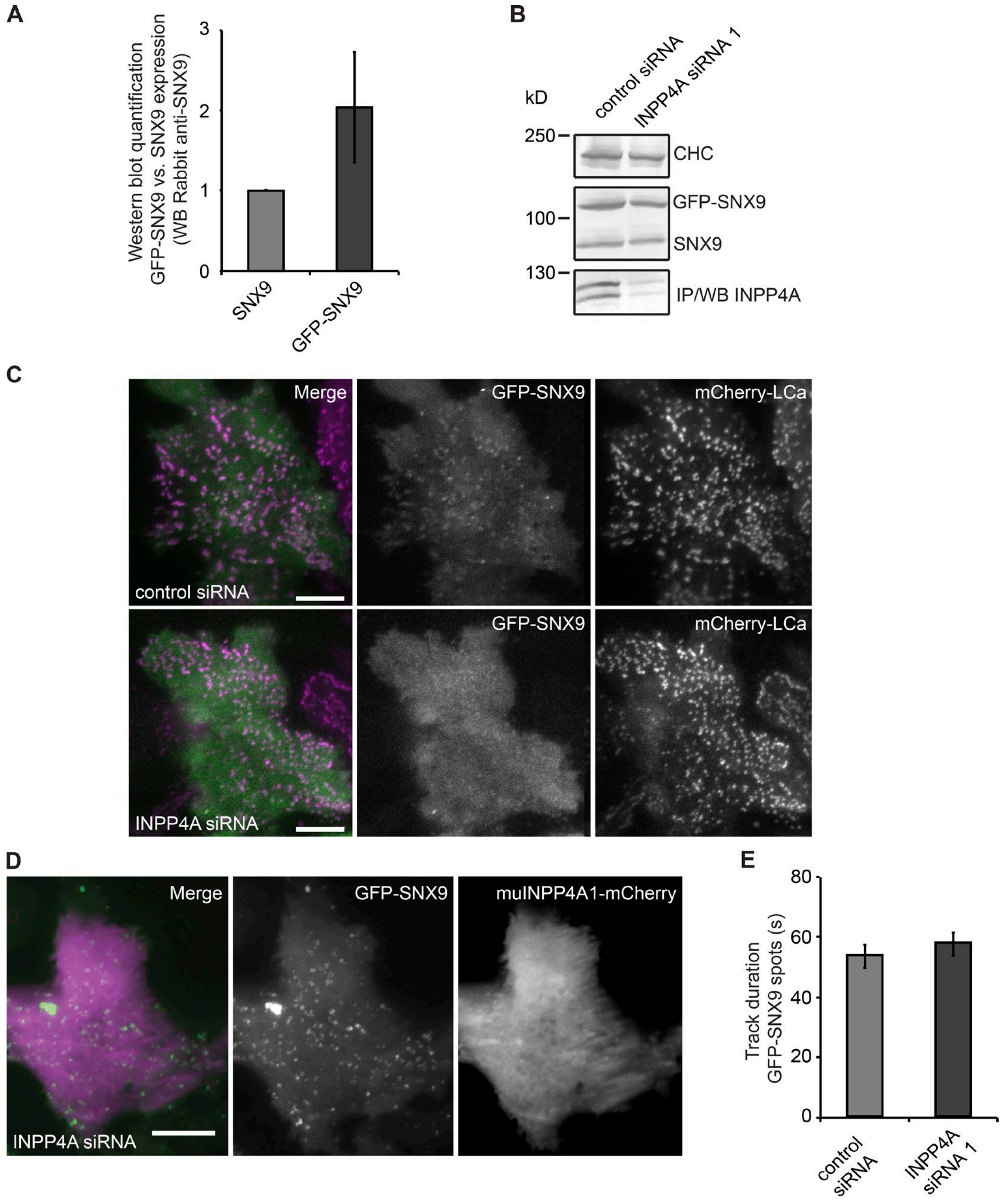

Figure S4. **Effect of INPP4A depletion on SNX9 during CME in HeLa cells stably expressing GFP-SNX9.** (A) Quantification of stable GFP-SNX9 expression in Flp-In TRex HeLa cells relative to endogenous SNX9 levels. Data show the mean of three independent experiments. Error bars are the SEM. (B) Western blot (WB) verifying levels of clathrin heavy chain, and endogenous and stably expressed GFP-SNX9 remain constant despite INPP4A depletion in HeLa cells. IP, immunoprecipitated. (C) Representative HeLa cells stably expressing GFP-SNX9 (green) and transiently expressing clathrin light chain a–mCherry (purple) treated with control or INPP4A siRNA. Images show maximal projected time frames from 4-min videos. SNX9 spots are reduced, whereas clathrin spots appear unaffected. (D) Transient expression of murine INPP4A-mCherry (purple) is diffusely localized in the cytoplasm and rescues GFP-SNX9 spots (green). Bars, 10 µm. (E) Duration of remaining SNX9 tracks is unchanged on INPP4A siRNA treatment.

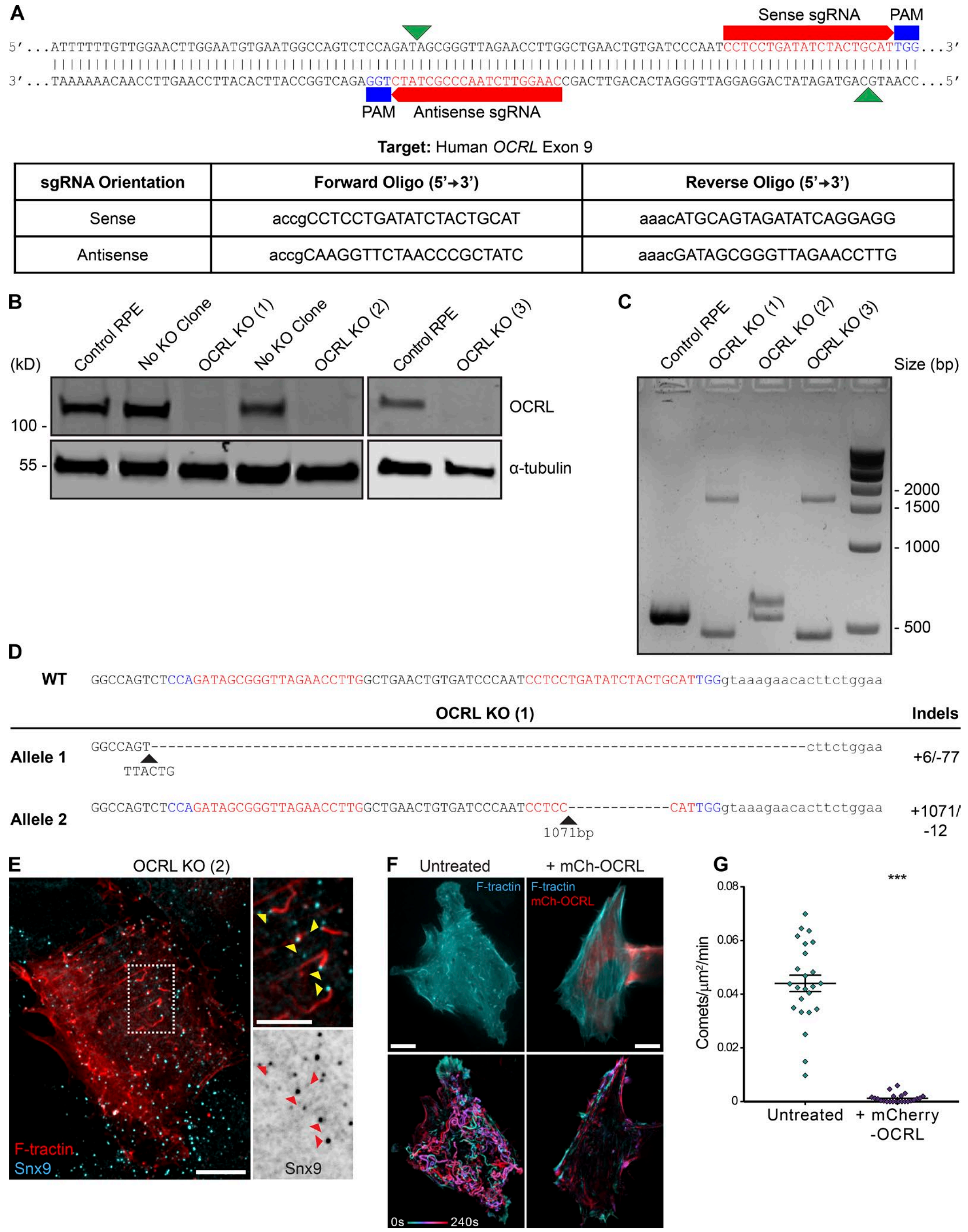

Figure S5. **Generation of RPE OCRL CRISPR KO cells.** (A) Sequence of *OCRL* exon 9 marked with the two sgRNA target sites (red) flanked by PAM regions (blue). Nick sites are marked by the green arrowheads. Lowercase letters on the sequences in the table indicate the restriction site overhangs for ligation into the all-in-one vector. Exon 9 is the first exon of the 5-phosphatase domain. It is 102 bp in length and contributes 34 aa to the protein. (B) Initial Western blot screen confirming OCRL KO in three out of five RPE CRISPR clones analyzed. α-Tubulin acts as a loading control. (C) PCR around the exon 9 target region confirming mutations in both alleles of the three CRISPR KO clones. Control PCR product, 538 bp. (D) Summary of sequencing data of the two mutant alleles of OCRL KO (1) compared with the WT control sequence. Red text indicates the sgRNA sites surrounded by the PAM sequences (blue text). Lowercase text indicates the sequence of the intron after exon 9. Sequence data show that allele 1 (the smaller of the two bands indicated in C) contains a small 6-bp insertion followed by a 77-bp deletion that continues beyond the end of the exon, eliminating the splicing site and continuing into the neighboring intron sequence. Allele 2 (the larger band observed in C) contains a large 1,071-bp insertion followed by a 12-bp deletion. Were these mutations expressed in a mutated protein product, both would produce a limited mutated amino acid sequence followed by an early stop codon. In allele 1, the first 11 aa of exon 9 would be followed by an additional 17 aa of the small insertion continuing into the intron before reaching a stop codon, whereas allele 2 would produce the first 28 aa of exon 9 followed by an additional 12 aa of the insert before reaching a stop codon. (E) Immunofluorescence of a second OCRL KO clone (OCRL KO 2) expressing F-tractin (red) and labeled for SNX9 (cyan, single channel in enlarged region). Consistent with previous observations, SNX9 puncta and actin comets with SNX9 localized at comet heads (arrowheads in enlarged region) are observed. (F) Live imaging over a 4-min period of F-tractin (cyan) in a representative untreated OCRL KO cell and a cell expressing murine mCherry-OCRL (red), as shown in Video 4, showing that motile actin comets in the KO are abolished on OCRL rescue. The single-channel image indicates a single time point, and the colored image shows a projection of the entire time course, temporally colored as indicated. Bars: (main images) 10 μm; (inset) 5 μm. (G) Quantification of actin comets from n = 24 (untreated)/23 (+mCh-OCRL) cell regions for each condition. Each data point is plotted individually, along with the mean ± SEM for each condition. Difference assessed by Student's *t* test: ***, $P < 0.0001$.

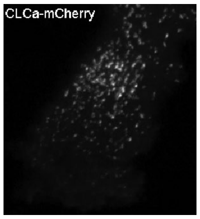

Video 1. **Time-lapse TIRFM imaging of representative Flp-In TRex HeLa cell expressing GFP-SNX9 (green) and clathrin-LaCa-mCherry (purple), as shown in Fig. 6 B.** SNX9 foci come and go on clathrin structures. Frames were taken at 800-ms intervals and replayed at 10 frames per second (fps); timescale in minutes. Bar, 10 µm.

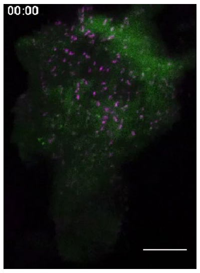

Video 2. **Time-lapse TIRFM imaging of INPP4A siRNA–treated Flp-In TRex HeLa cells expressing GFP-SNX9 (green) and clathrin-LaCa-mCherry (purple), as shown in Fig. 6 B.** SNX9 recruitment to clathrin is inhibited. Frames were taken at 800-ms intervals and replayed at 10 fps; timescale in minutes. Bar, 10 µm.

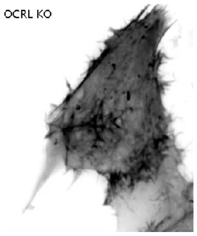

Video 3. **Time-lapse spinning disk confocal imaging of representative control RPE and OCRL KO (1) cells expressing mNeon-Green–F-tractin, as shown in Fig. 7 A.** Note the comets present in the KO cells. Frames were taken every 2 s for 4 min and are replayed at 10 fps. Bar, 10 µm.

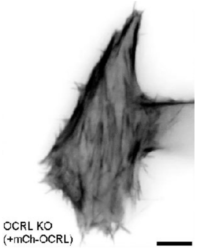

Video 4. **Time-lapse spinning disk confocal imaging of mNeonGreen–F-tractin in representative OCRL KO (1) cells left untreated or cotransfected with a murine mCherry-OCRL rescue construct, as shown in Fig. S5 F.** Note that comets are abolished after rescue with the OCRL construct. Frames were taken every 2 s for 4 min and are replayed at 10 fps. Bar, 10 µm.

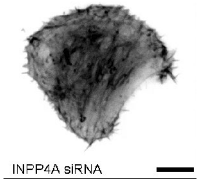

Video 5. **Time-lapse spinning disk confocal imaging of representative OCRL KO (1) cells expressing mNeonGreen–F-tractin and treated with nontargeting control siRNA or INPP4A siRNA (1), as shown in Fig. 7 F.** Note the reduction in comets after INPP4A depletion. Frames were taken every 2 s for 4 min and are replayed at 10 fps. Bar, 10 µm.

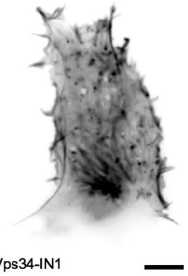

Video 6. **Time-lapse spinning disk confocal imaging of representative OCRL KO (1) cells expressing tomato–F-tractin and treated with DMSO (1:1,000), 2 μM wortmannin, or 10 μM Vps34-IN1 in serum-free media for 1 h before imaging, as shown in Fig. 8 B.** Note the reduction in comets after treatment with either inhibitor compared with the DMSO control. Frames were taken every 2 s for 4 min and are replayed at 10 fps. Bar, 10 μm.
